# Impact of Healthy Aging on Multifractal Hemodynamic Fluctuations in the Human Prefrontal Cortex

**DOI:** 10.3389/fphys.2018.01072

**Published:** 2018-08-10

**Authors:** Peter Mukli, Zoltan Nagy, Frigyes S. Racz, Peter Herman, Andras Eke

**Affiliations:** ^1^Institute of Clinical Experimental Research, Semmelweis University, Budapest, Hungary; ^2^Department of Physiology, Semmelweis University, Budapest, Hungary; ^3^Department of Radiology and Biomedical Imaging, Yale University, New Haven, CT, United States

**Keywords:** aging, cerebral hemodynamics, neurovascular coupling, near-infrared spectroscopy (NIRS), correlation-based signal improvement, multifractality, multifractal analysis, signal summation conversion

## Abstract

Fluctuations in resting-state cerebral hemodynamics show scale-free behavior over two distinct scaling ranges. Changes in such bimodal (multi) fractal pattern give insight to altered cerebrovascular or neural function. Our main goal was to assess the distribution of local scale-free properties characterizing cerebral hemodynamics and to disentangle the influence of aging on these multifractal parameters. To this end, we obtained extended resting-state records (*N* = 2^14^) of oxyhemoglobin (HbO), deoxyhemoglobin (HbR) and total hemoglobin (HbT) concentration time series with continuous-wave near-infrared spectroscopy technology from the brain cortex. 52 healthy volunteers were enrolled in this study: 24 young (30.6 ± 8.2 years), and 28 elderly (60.5 ± 12.0 years) subjects. Using screening tests on power-law, multifractal noise, and shuffled data sets we evaluated the presence of true multifractal hemodynamics reflecting long-range correlation (LRC). Subsequently, scaling-range adaptive bimodal signal summation conversion (SSC) was performed based on standard deviation (σ) of signal windows across a range of temporal scales (*s*). Building on moments of different order (*q*) of the measure, σ(*s*), multifractal SSC yielded generalized Hurst exponent function, *H*(*q*), and singularity spectrum, *D*(*h*) separately for a fast and slow component (the latter dominating the highest temporal scales). Parameters were calculated reflecting the estimated measure at *s* = *N* (focus), degree of LRC [Hurst exponent, *H*(2) and maximal Hölder exponent, *h*_max_] and measuring strength of multifractality [full-width-half-maximum of *D*(*h*) and Δ*H*_15_ = *H*(−15)−*H*(15)]. Correlation-based signal improvement (CBSI) enhanced our signal in terms of interpreting changes due to neural activity or local/systemic hemodynamic influences. We characterized the HbO-HbR relationship with the aid of fractal scale-wise correlation coefficient, *r*_σ_(*s*) and SSC-based multifractal covariance analysis. In the majority of subjects, cerebral hemodynamic fluctuations proved bimodal multifractal. In case of slow component of raw HbT, *h*_max_, and *Ĥ*(2) were lower in the young group explained by a significantly increased *r*_σ_(*s*) among elderly at high temporal scales. Regarding the fast component of CBSI-pretreated HbT and that of HbO-HbR covariance, *h*_max_, and focus were decreased in the elderly group. These observations suggest an attenuation of neurovascular coupling reflected by a decreased autocorrelation of the neuronal component concomitant with an accompanying increased autocorrelation of the non-neuronal component in the elderly group.

## Introduction

Scale-free dynamics is an ubiquitous property of physiological processes (West, 1991; Eke et al., 2000; Ivanov et al., 2001) such as low frequency fluctuations of cerebral hemodynamics (Fox and Raichle, 2007; Herman et al., 2009; Pierro et al., 2012) and neural activity (Linkenkaer-Hansen et al., 2001; Ivanov et al., 2009; He, 2014). Scale-free dynamics is a hallmark of *complexity* viewed as an emergent property of biological systems composed of numerous elements with a network of stochastic (typically weak) connections amongst them (Csermely, 2006). Several human studies investigated the scale-free phenomenon of functional brain imaging signals by using mono-(Eke and Hermán, 1999; Thurner et al., 2003; Maxim et al., 2005; Eke et al., 2006; Khoa and Nakagawa, 2008; Bullmore et al., 2009; He et al., 2010; He, 2011; Herman et al., 2011) and multifractal analysis (Shimizu et al., 2004; Wink et al., 2008; Ciuciu et al., 2012; Quang Dang Khoa and Van Toi, 2012). Monofractal analysis reveals *global*, long-range correlation (LRC) structuring in terms of the influence of past events in the process on future ones (Bassingthwaighte et al., 1994; Eke et al., 2000, 2002). Multifractal analysis yields a distribution of fractality measures (Barabási et al., 1991; Stanley et al., 1999; Kantelhardt et al., 2002; Ihlen and Vereijken, 2010; Mukli et al., 2015) that enables a more detailed characterization of *local* temporal scaling behavior provided that fluctuations at wide range of temporal scales are sufficiently represented in the sampled physiological process (Eke et al., 2012). The estimation of these *complexity parameters* is essentially based on a power-law model fitted to the appropriate statistics of the data, which is reliable only if sample size is large enough [at the order of hundreds, Eke et al., 2002; Clauset et al., 2009]. Such statistics usually shows power-law scaling—indicating LRC—within a bounded interval of temporal scales usually termed as scaling range (SR; Caccia et al., 1997). In addition to the finite representation of the dynamics the lower and upper boundary of SR are determined by the signal genesis of the underlying physiological process. Nevertheless, numerous examples of empirical data exhibit multiple SR indicating *multimodal scaling*, see examples cited in Nagy et al. (2017). Multimodality has also been of concern in case of cerebral hemodynamics and typically present itself with two (case of bimodality) or even more apparent SRs in which the statistical measure of fractal analysis scales with a different exponent (Nagy et al., 2017).

Application of a possible bimodal scale-free model on resting-state hemodynamic signals requires a measurement technology, which assures that the process is sampled at high enough rate in long enough records. Near-infrared spectroscopy (NIRS) is an emergent imaging technology which readily captures cerebrocortical resting-state hemodynamic fluctuations at a cm spatial resolution and at high sampling frequency with no particular limitations on signal length (Jöbsis, 1977; Chance et al., 2007; Fox and Raichle, 2007). In case of continuous wave near-infrared spectroscopy (cwNIRS), the measured intensity signals are determined by the relative tissue concentration of total hemoglobin (HbT) and its constituents: oxy- and deoxyhemoglobin (HbO and HbR, respectively; Cope et al., 1988).

By now the physiological underpinnings of the functional NIRS (fNIRS) signal has been elucidated (Jöbsis, 1977; Kocsis et al., 2006a; Tachtsidis et al., 2008). As to its dynamics, oscillations of cerebral hemodynamics has been characterized by spectral analysis of NIRS signals (Elwell et al., 1999). Monofractal pattern of NIRS spectral data was first reported by Eke and Herman for the human brain cortex (Eke and Hermán, 1999). Later, multifractal properties of fNIRS were also demonstrated (Quang Dang Khoa and Van Toi, 2012). These pioneering reports understandably focused on signal analysis and not making an attempt to identify the underlying physiological mechanisms shaping the observed complex patterns. As to the nature of local hemodynamic fluctuations, they are primarily elicited by neural activity via neurovascular coupling (NVC; Devor et al., 2003; Drake and Iadecola, 2007) but the hemodynamic response is also modulated by endothelial mechanism (Li et al., 2013; Chen et al., 2014). In addition, systemic hemodynamic effects should be considered (Yamada et al., 2012; Scholkmann et al., 2014) for an enhanced interpretation of results obtained from resting-state records from subjects with similar age. Apart from non-biological noise and motion artifacts, resting-state NIRS (rsNIRS) signal bears the fingerprint about systemic hemodynamics such as cardiac cycle and respiration (Tian et al., 2009; Li et al., 2013). Separation of the functional and the systemic components became of considerable interest and various approaches have been developed to address this issue (Scholkmann et al., 2014). Correlation between the fluctuations of oxy- and deoxyhemoglobin concentration is the basis of signal improvement presented by Cui et al. (2010) and the technique proposed by Yamada et al. (2012). Under certain assumptions—that usually hold in resting state, neural activity renders the relationship between HbO and HbR fluctuations more anticorrelated while fluctuations of systemic origin in the resting state (Cui et al., 2010; Scholkmann et al., 2014) has a correlated influence on hemoglobin chromophores.

The nonstationary fractal character of HbT (Eke et al., 2006) implies that its constituents, HbO, and HbR, also exhibit non-stationary dynamics. Consequently, their relationship should be explored in terms of a non-stationary characterization of correlation. Therefore, the HbO-HbR relationship was studied with the aid of scale-wise fractal cross-correlation coefficient and a novel adaptation of multifractal covariance analysis. The former is essentially a measure applicable to nonstationary time series building on the correlation of scale-wise mean variances obtained separately for HbO and HbR signal (Podobnik and Stanley, 2008; Zhou, 2008), while the latter is a multifractal approach examining the scaling properties—and its moments of various order—of HbO-HbR covariance similarly to the analyses described in Refs (Kristoufek, 2011; Jiang et al., 2016; Zhao et al., 2017).

It has been shown that aging (Goldberger et al., 2002; Lipsitz, 2003) and various diseases (Ivanov et al., 1999; Goldberger et al., 2002; Maxim et al., 2005) affect complexity parameters and the impact of other factors, such as gender (Ni et al., 2014), have also been recognized. This study contributes to this accumulating body of knowledge on the influence of aging on the complexity of physiological processes. In general, the contraction of homeodynamic space is an essential attribute of an aging biological system meaning that the dynamics of physiological processes in an elderly person is typically confined to a restricted state space (Rattan, 2014). Taking the cardiovascular system as an example the well-known dependence of heart rate variability (Beckers et al., 2006; Vandeput et al., 2012) on age can be attributed to a decline in autonomic modulation (Nunes Amaral et al., 2001; Lipsitz, 2003; Tulppo et al., 2005; Silva et al., 2017) reducing the adaptational reserve of regulatory mechanisms (Goldberger et al., 2002). Such reports on aging and altered complexity parameters are typically based on demonstrating coincidences, while there is a palpable need to establish a causal relationship for the changing complexity.

Accordingly, our goal was to provide plausible explanation for the physiological mechanism of observed age-related alterations based on parameters of complexity obtained from measures of brain hemodynamics captured by rsNIRS. Firstly, the measured signals were evaluated for the presence of true LRC-type multifractality. Multifractal parameters obtained for young and elderly volunteers were compared to assess the impact of aging on the complexity of cerebral hemodynamics. In addition, we extended our analysis incorporating the oxy- and deoxyhemoglobin signals in order to explore the age-dependent alterations in their relationship. The influence of age was characterized by exploring the strength of causal link between these measures of the coupled fluctuations of oxy- and deoxyhemoglobin and multifractal parameters of cerebral hemodynamics.

## Methods

Extended records of fluctuating rsNIRS signals from the human brain prefrontal cortex (PFC) analyzed for their multifractality in this work have been collected in a previous study of the group reporting on the monofractal serial correlation in these signals (Eke et al., 2006).

### Near infrared spectroscopy

According to the principle of cwNIRS, backscattered light intensities were measured at wavelengths of 775, 830, 849, and 907 nm by a NIRO 500 Cerebral Oxygen Monitor (Hamamatsu Photonics, Hersching, Germany), a single-channel instrument. The mean penetration depth of near infrared photons for the 4 cm interoptode distance of this device can be estimated at approximately 2 cm (Firbank et al., 1998), thus our NIRS optode sampled the brain cortex (Chance, 1994). Based on the modified Beer-Lambert law (Kocsis et al., 2006b) the relative tissue concentrations of HbO and HbR were calculated along with HbT obtained as their sum. While the fluctuation of HbT reflects on cerebral blood volume (CBV) dynamics, that of the other two chromophores and their relationship are dependent on oxygenation, too.

### Data collection

HbO, HbR, and HbT data were dumped via the RS232 port of the NIRO instrument into a computer file at a rate of 2 Hz. Extended records of HbO, HbR, and HbT samples were created and processed for each subject in length of *N* = 2^14^ proven to be adequate for fractal analysis (Eke et al., 2002). The source and detector fibers were secured in a rubber pad. The optode was mounted just under the hairline over the forehead. The cranium was shielded from ambient light. Instrument noise was determined by placing the optode over a slab of “mock” brain, whose scattering (μ_s_ = 10.96 1/mm) and absorption (μ_a_ = 0.099 1/mm) coefficients were matched to that of the human brain (courtesy of Prof. Britton Chance, University of Pennsylvania, Philadelphia, U.S.A.). The power of instrument noise was found negligible when compared to the power of resting-state fluctuations *in vivo* (Eke et al., 2006).

### Subjects

Following approval by the Local Research Ethics Committee of Semmelweis University and having obtained informed written consent, 52 healthy volunteers with no neurological disorders (28 women, 24 men) participated in the study. To evaluate the effect of age and gender, subjects were assigned into groups of young females (Y_F_, *n* = 9, age < 45 years), young males (Y_M_, *n* = 13, age < 45 years) elderly females (E_F_, *n* = 19, age≥45 years) and elderly males (E_M_, *n* = 10, age≥45 years). The rsNIRS measurements were carried out in a comfortable sitting position in a session slightly exceeding 2.5 h as previously described in (Eke et al., 2006).

### Data preprocessing

Multifractal analyses were performed on raw signals and following correlation-based signal improvement (CBSI; Cui et al., 2010; Scholkmann et al., 2014), the latter designed to remove correlated (systemic) influences (e.g., head movement). Specifically, this preprocessing step incorporate a standard deviation ratio of HbO and HbR that is used in their subsequent linear combination yielding:  CBSIHbT=12 ​​⋅(HbR−(σO(N)/σR(N))⋅HbR)+12​ ⋅(HbO−(σR(N)/σO(N))⋅HbO). The improved HbT signal is regarded as a representation of anticorrelated hemodynamics attributable to local hemodynamic influences and oxygen consumption accompanying neuronal activity.

### Multifractal analyses

#### Multifractal scaling analysis

##### Signal summation conversion (SSC) method

**Figure 1:**
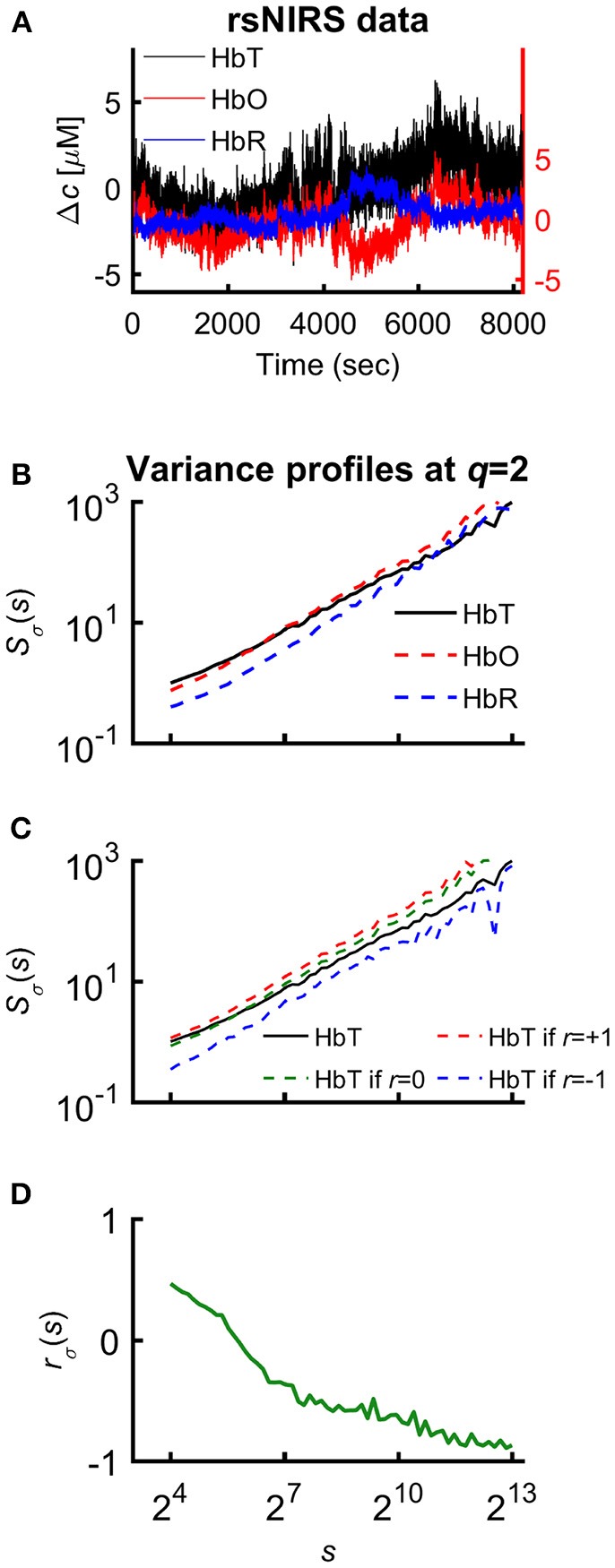
Various representations of the measured NIRS signals. **(A)** Resting-state raw NIRS signal components. **(B)** Variance profiles of different signal components. **(C)** Variance profiles of HbT calculated at different set cross-correlation levels. **(D)** Fractal correlation coefficient between HbO and HbR as a function of scale. In this paper multifractal scaling function values are actually given by *S*_σ_(*q, s*) [or by *S*_σ_(*s*) for *q* = 2], where the subscript σ refers to the measure of the chosen multifractal method (SSC).

The multifractal scaling functions of HbT, HbO, and HbR were calculated by the multifractal SSC method (Mukli et al., 2015) as the basis of our approach to evaluate the multifractality of our bimodal rsNIRS signals (Figure 1). For detailed description of the MF-SSC method the reader is referred to Refs. (Mukli et al., 2015; Nagy et al., 2017). Briefly, multifractal SSC uses a measure depending on the temporal scale (*s*) of signal window—bridge-detrended variance σ(*s*), see Ref. (Eke et al., 2000)—and a set of *q*-order statistical moments, to create corresponding moment-wise generalized variance profiles, *S*_σ_(*q, s*) of Equation 1, spanning across a range of temporal scales within the chosen analytical SR.


(1) $$\begin{array}{r} S_{\sigma }\left(q,s\right)={\left[\frac{1}{N_{s}}\overset{N_{s}}{\underset{v=1}{\sum }}\mu_{v}^{q}\left(s\right)\right]}^{1/q}\propto s^{H\left(q\right)} \end{array}$$

Specifically, 60 logarithmically spaced time scales were chosen between *s*_*min*_ = 16 and *s*_*max*_ = 8192 for computing σ_*v*_(*s*) in each non-overlapping time window [*v* = 1, 2, …, *N*_*s*_ = int(*N*/*s*)] of cumulatively summed signal. The low temporal scales dominated by fast fluctuations with weak, non-fractal autocorrelation (Eke et al., 2006) were excluded and the fairly high scales (Nagy et al., 2017) with well-manifested scale-free processes were secured. The selected moment orders ranging from −15 to 15 were adequate^1^ to capture both large- and small-variance dynamics in a fluctuating rsNIRS signal; the former being emphasized by variance profiles corresponding to positive moments, the latter by those corresponding to negative moments (Grech and Pamuła, 2012; Mukli et al., 2015). Please note that statistical estimates obtained at *q* = 2 obey rules of *linearity*, while in fact, those for *q*≠2 capture *non-linearity* in system dynamics (Gómez-Extremera et al., 2016; Bernaola-Galván et al., 2017).

##### Relationship between variance profiles of hemoglobin chromophores

Since HbT = HbO+HbR, it is the generalized Bienaymé formula (Equation 2) which describes the relationship between their variance profiles^2^: ^T^*S*_σ_(2, *s*), ^O^*S*_σ_(2, *s*), and ^R^*S*_σ_(2, *s*) in an exact form^3^ (Nagy et al., 2017).


(2) STσ(2,s)2=SOσ(2,s)2+SRσ(2,s)2+2rσ(s)·SOσ(2,s)·SRσ(2,s).

The unknown factor is the scale-wise fractal correlation coefficient, *r*_σ_(*s*); all the others are scaling function values directly calculated from the measured signal.

#### Focus-based scaling-range adaptive analyses

Focus-based multifractal analysis (FMF-SSC) were carried out (Mukli et al., 2015) to estimate the generalized Hurst exponent, *H*(*q*), which is essentially a set of slopes of the scaling function profiles fitted in the analytical SR, as expressed by


(3) $$\begin{array}{r} S_{\sigma }\left(q,s\right)\propto s^{H\left(q\right)}. \end{array}$$

*H*(*q*) describes the moment-wise or *global* distribution of fractal correlation (essentially that of the fractal dimension) in the signal, thereby generalizing *H*(2), the usual outcome of monofractal time series analysis. Taking *H*(*q*) as its input, the multifractal formalism (Frisch and Parisi, 1985; Barabási and Vicsek, 1991; Muzy et al., 1993; Eke et al., 2012) via the multiscaling exponent, τ(*q*) = *qH*(*q*)−1, and Legendre transformation will yield *D*(*h*), the *local* distribution of the fractal dimension or singularity strength, *D*, as a function of roughness or Hölder exponent, *h*.


(4) $$\begin{array}{rcl} h & = & \frac{d\tau \left(q\right)}{dq}, \end{array}$$

(5) $$D\left(h\right)=\underset{q}{\text{inf}}\left(qh-\tau (q)\right).$$

Incorporating the focus—obtained as a fitted intersection of scaling function profiles at *s* = *N*–ensures monotonously decreasing *Ĥ*(*q*) and thus stable, uncorrupted *D*(*h*). Enforcing the focus of scaling function, ln(Ŝ_σ_(*N*)), when regressing for *Ĥ*(*q*) was recognized as a prerequisite to obtain stable results of multifractal analysis (Mukli et al., 2015).

**Figure 2:**
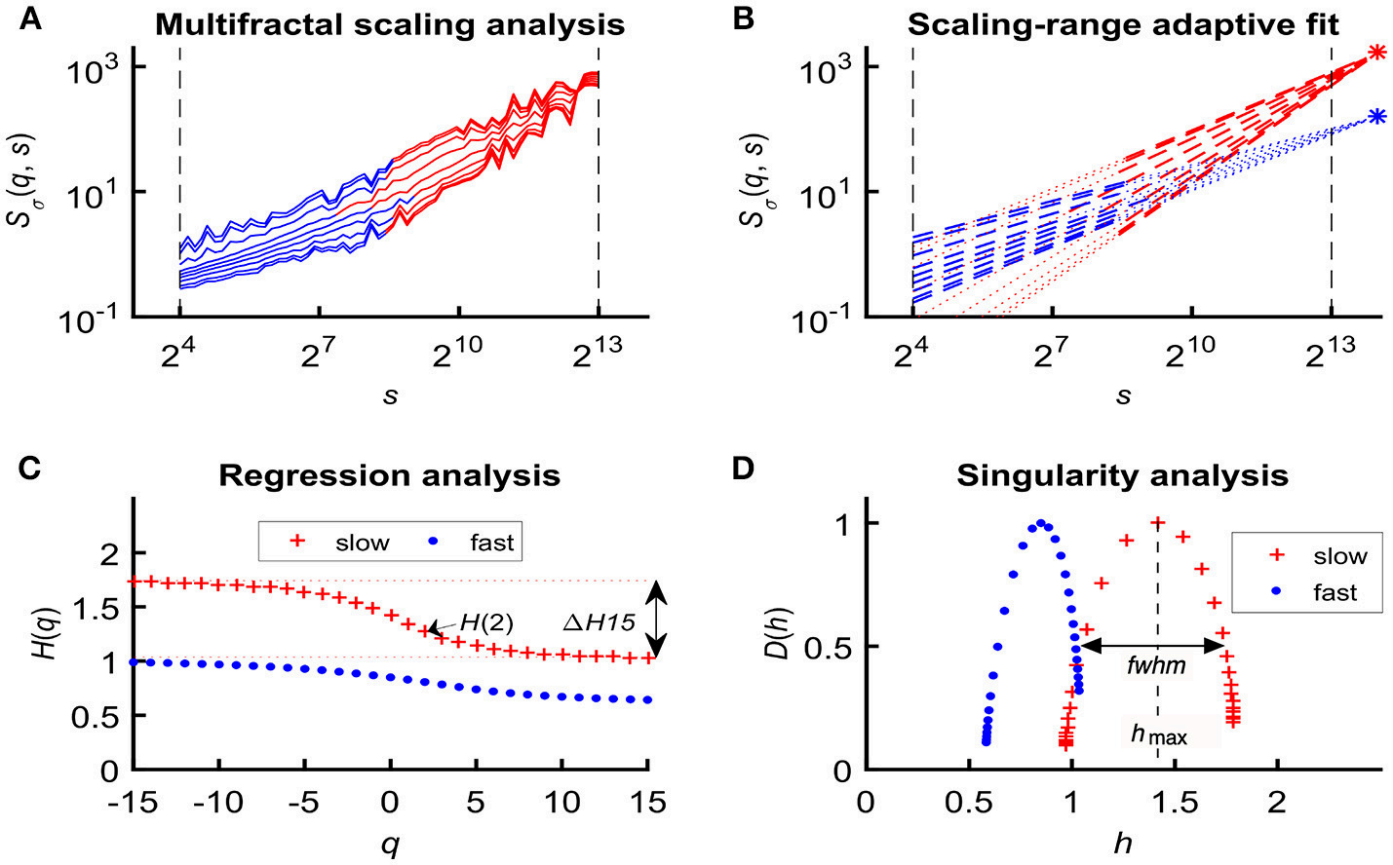
Steps of bimodal multifractal SSC analysis. **(A)** Scaling function of SSC as moment-wise generalization of variance profiles. Separate components are marked in blue (fast) and red (slow). **(B)** The sets of power-law functions fitted separately for the two components with focus-based regression. **(C)** Generalized Hurst exponent functions, *H*(*q*)s of the two components as functions of moment order *q*. The focus point and *H*(*q*) for both components (*f* , fast; *s*, slow) were iterated for finding the scale with minimum SSE(*s'*(*q*)) as the true breakpoint at a given moment, which process finally yields ln (^*s*^Ŝ_σ_(*N*)), ln (^*f*^Ŝ_σ_(*N*)), ^*s*^*Ĥ*(*q*), and ^*f*^*Ĥ*(*q*) with the best fit. **(D)** Singularity spectra of the two components. *Multifractal endpoint parameters*: the highest singularity strength (*D* = 1) is associated with the maximal Hölder exponent (*h*_max_), which usually correlates well with *H*(2), a measure of global LRC in the signal. Distribution of local scale-free behavior is captured in Δ*H*_15_ = *H*(−15) – *H*(15) and in full-width at half maximum (*fwhm*) of *D*(*h*) respectively, reflecting degree of multifractality.

In an attempt to find the best fitting model for the observed scaling functions we adapted the concept of bimodality originally described in the frequency domain (Eke et al., 2006). This pattern can be recognized as two scale-free processes separated by moment-wise breakpoint scales (*s*'(*q*)) (Nagy et al., 2017). The less correlated (“fast”) component dominates the lower range of scales while a more correlated (“slow”) component is characteristic in the higher scales (Figure 2A). A breakpoint-based bilinear regression model (denoted as moment-wise SR adaptive) was implemented as described in Ref. (Nagy et al., 2017). Briefly, it is an iterative process by estimating breakpoint scales that minimize sum of squared error (SSE) of the residuals for each and every moment as


(6) SSE(s′(q))=∑q=−1515[∑s=smins′(q)(H^f(q)·(lns−lnN)                   +lnS^fσ(N)−lnSσ(q,s))2+ (H^f(q)·(lns−lnN)                          +lnS^sσ(N)−lnSσ(q,s))2],

where ln(^*s*^Ŝ_σ_(*N*)) and ln(^*f*^Ŝ_σ_(*N*)) are the moment-independent foci (Figure 2B) associated with the slow and fast components, respectively. ^*s*^*Ĥ*(*q*), ^*f*^*Ĥ*(*q*) are the slopes (Figure 2C) of the fitted two lines of regression^4^ (i.e., the generalized Hurst exponent functions of the two components). This iteration thus adaptively yields the best segregation of scaling ranges. $\hat{H}$(*q*) and *D*(*h*) (Figure 2D) are obtained for the fast and the slow component, respectively.

We calculated global multifractal endpoint parameters to characterize the degree of autocorrelation [maximal Hölder exponent, *h*_max_ and monofractal Hurst exponent, $\hat{H}$(2)] and multifractality [Δ*H*_15_ = *H*(−15)–*H*(15), and full-width at half maximum (*fwhm*) of *D*(*h*) (Wink et al., 2008; Grech and Pamuła, 2012)] in the measured cerebral hemodynamic signals as illustrated in lower panels of Figure 2.

#### Evaluating true multifractality

Since multifractal tools—including FMF-SSC—readily produce seemingly realistic values for multifractal measures such as *D*(*h*) even in the absence of true multifractality (Kantelhardt et al., 2002; Clauset et al., 2009; Grech and Pamuła, 2012); it is mandatory to evaluate our empirical signals in this regard using the following framework. Accordingly, because our FMF-SSC method always produces uncorrupted *D*(*h*) irrespective whether the signal is a true multifractal or not, this property needs to be tested separately (Figure A1). Verification of true multifractality consists of three subsequent steps: (*i*) identifying general scale-free behavior with power spectral density (PSD) analysis, (*ii*) distinguishing true multifractality from multifractal noise, and (*iii*) determining the origin of the expressed multifractal scaling. Therefore, in these tests, true, long-range correlated multifractals are to be distinguished from processes lacking scale-free properties or not showing autocorrelation. Failing to pass in any of the aforementioned tests resulted in the exclusion of the subject in question from further analysis. Details of this framework are explained in the Appendix (Supplementary Material) and in Ref. (Racz et al., 2018).

#### Characterizing HbO-HbR relationship

##### Scale-wise fractal cross-correlation coefficient

One approach to assess the relationship of HbO and HbR fluctuations is to calculate a measure of cross-correlation by using variance profiles. After rearrangement of Equation 2 it is possible to express *r*_σ_(*s*):


(7) rσ(s)=STσ(2,s)2−SOσ(2,s)2−SRσ(2,s)22·SOσ(2,s)·SRσ(2,s).

This measure indicates whether the fluctuations at a given scale are enhanced (*r*_σ_(*s*) > 0), diminished (*r*_σ_(*s*) < 0) by coupling HbO and HbR signal or their relationship is insensitive to coupling between them (*r*_σ_(*s*) = 0).

The fractal cross-correlation analysis yielding *r*_σ_(*s*) is strikingly similar—in terms of calculation steps and order—to the detrended cross correlation analysis, the major difference of these methods concerns their measure (Podobnik and Stanley, 2008). The cited approach uses fluctuation while ours calculates bridge-detrended variance to assess correlation of coupled non-stationary time series. When comparing the above scale-wise approaches with the standard means of calculating cross-correlation (i.e., Pearson and Spearman), the first major difference is that the prerequisite of stationarity applies to the latter methods. Furthermore, the sequence of calculation steps is critical, too: because when the standard cross-correlation is calculated it is followed by a step of averaging effectively abolishing the scale-wise information. It is worth of noting that Spearman proved a more robust standard measure of correlation than the Pearson coefficient as the latter assumes not only stationarity but linearity, too (Zimeo Morais et al., 2018).

##### Multifractal covariance analysis

The other approach is based on the extension of FMF-SSC in order to analyze the scaling of long-range cross-correlation. This method assesses the multifractality emerging genuinely from coupled oxy- and deoxyhemoglobin fluctuations. Scale- and moment-wise bridge-detrended covariances (_Cov_) were calculated of HbO and HbR signals to yield an estimate of bivariate generalized Hurst exponent function, ^OR^*H*(*q*).


(8)  ORSCov(q,s)=[1Ns∑v=1Ns|Cov|ORvq(s)]1/q∝sHOR(q)

Now applying Equations 3–5 yields the corresponding singularity spectrum and multifractal endpoint parameters. Covariance truly scales only if the ^OR^*Ĥ*(*q*) function differs from the average of ^R^*Ĥ*(*q*) and ^O^*Ĥ*(*q*). Therefore this comparison must be carried out after obtaining the distribution of scaling exponents and output parameters of multifractal analysis. Prior to that, moment-wise bimodal regression analysis had been performed on the *q*-wise (generalized) variance profiles of HbO, HbR, and HbT and on the HbO-HbR covariance profile in the same manner.

### Descriptive statistical analyses

Normal distributions in each independent sample were checked by Shapiro-Wilk's test. Difference between group means or medians were considered significant in case of *p* < α_s_, α_s_ = 0.05 (level of significance). Homogeneity of variances was confirmed by Levene's test.

#### Assessing the effect of age and gender

Two-way univariate ANOVA were performed treating multifractal endpoint parameters and *r*_σ_(*s*) as dependent variables and assuming that there was an interaction between age and gender (categorical factors). Most of the results presented in this paper are based on the output of two-way ANOVA with Tukey's *post hoc* test. Had the prerequisites of ANOVA been not met, group means were compared with two-sample *t*-test (with Welch's correction for inhomogeneous variances in case of significant Levene's test). In the absence of normal distribution, the comparison of group medians was performed by Mann-Whitney U test.

Multivariate ANOVA (MANOVA) was performed to detect age- and gender-related differences between direct descriptors of the scaling function: ^*s*^*Ĥ*(2), ^*f*^*Ĥ*(2), ln(^*s*^Ŝ_σ_(*N*)), ln(^*f*^Ŝ_σ_(*N*)). As changes occurring by coincidence is of concern, MANOVA tests were used taking *Ĥ*(2), ln(^*s*^Ŝ_σ_(*N*)), ln(^*f*^Ŝ_σ_(*N*)) and *r*_σ_(*s*) (at scales corresponding to the slow component *s* = 4,344, 4,828, 5,367, and 5,965) as dependent variables. The *p* < 0.05 of Wilks's test suggests that the same subjects are responsible for each of the between-group differences. Finally, examining the degree of correlation of dependent variables (expressed as *r*^2^) enables to identify the most relevant endpoint parameters of multifractal analysis.

#### Explaining the variance profiles of HbT

In order to identify a link between the altered scale-wise fractal cross-correlation coefficient and the altered multifractal endpoint parameters, the influence of *r*_σ_(*s*) as an independent variable were evaluated on ^T^*S*_σ_(2, *s*) in a general linear model (GLM). The Bienaymé formula expresses an explicit relationship between ^T^*S*_σ_(2, *s*) and the scaling function values obtained for HbO, HbR, and *r*_σ_(*s*) (Equation 7). Accordingly, the variability of ^T^*S*_σ_(2, *s*) were explained with the aid of multiple regression tests that were performed for each temporal scales. The regressors of this model were ^O^*S*_σ_(*2, s*), ^R^*S*_σ_(*2, s*), and *r*_σ_(*s*) but categorical predictors (age and gender) were not included.

Subsequently, – in addition to the regressors of the previously described test—we took into account the effect of age and gender by applying scale-wise covariance analysis (AnCova). Specifically, age and gender were treated as categorical factors and ^O^*S*_σ_(*2, s*), ^R^*S*_σ_(*2, s*), and *r*_σ_(*s*) as covariates. Before AnCova, homogeneity of slopes model was evaluated for each *s*, significant result of this test means that AnCova is inapplicable due to an interaction between categorical predictors and covariates. In these cases the separate slopes model was used which include these interaction terms.

### Software

For a more detailed description of our analytical flowchart as a guide for the FMF-SSC analysis, see (Eke et al., 2000, 2012; Mukli et al., 2015; Nagy et al., 2017). The above aspects of multifractal analyses of rsNIRS time series have been implemented in Matlab 2012 (The MathWorks, Inc., Natick, MA, U.S.A.) by custom scripts written by the authors based on the recently published “MultiFracTool” software (Mukli et al., 2015; Nagy et al., 2017; Racz et al., 2018). The toolbox can be requested from the corresponding author. Statistical analyses were performed with StatSoft Statistica 13.2.

## Results

### The presence of true bimodal multifractality

All measured signals showed an apparent bimodal scaling function. True multifractality was confirmed in 44 subjects, two of them with an unacceptable fit of the bimodal model. For further details, see Appendix in Supplementary Material.

### Impact of age-and gender on multifractal endpoint parameters

**Figure 3:**
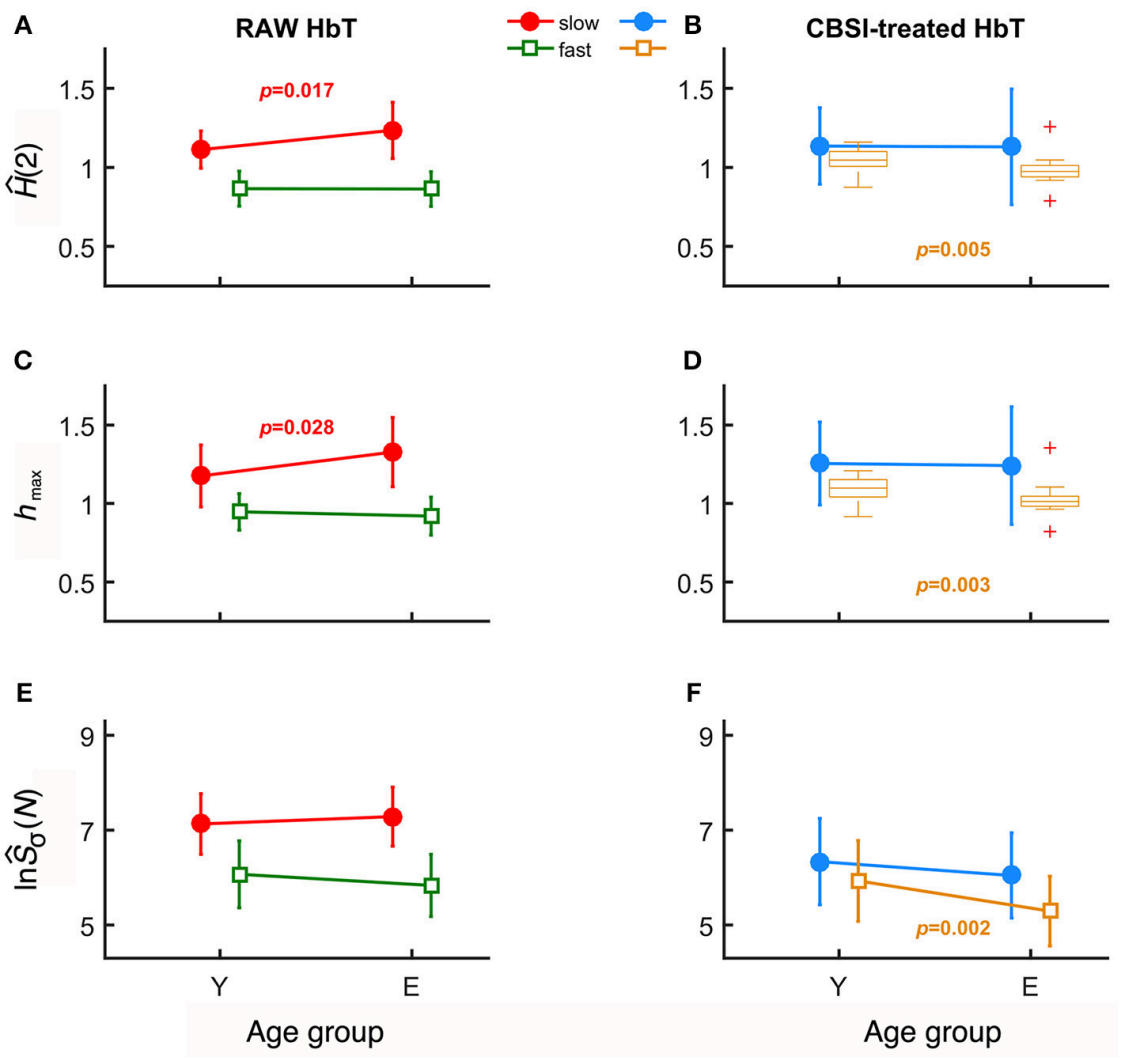
Results of univariate statistical analysis of multifractal endpoint parameters. *Ĥ*(2) of slow and fast components in the two age groups calculated from raw **(A)** and on CBSI-pretreated **(B)** HbT signals. *h*_max_ obtained from raw **(C)** and CBSI-treated **(D)** HbT signals. Focus of raw **(E)** and CBSI-pretreated **(F)** HbT signals. Recall that the raw HbT signal represents neuronal and non-neuronal events combined, while the CBSI-pretreated signal is a enhanced representation of the underlying neurodynamics. Accordingly, the fact that we found significant differences in the slow component for the raw HbT signal and in the fast component for the CBSI-pretreated signals identifies the slow component emerging dominantly from vascular events, while the fast component attributed mainly to neurodynamics.

In case of raw HbT signals, the degree of autocorrelation for the slow component, marked by ^*s*^*Ĥ*(2), significantly increased with age unlike for the fast component (Figure 3A). Conversely, the neural component obtained with CBSI significantly decreased with age for the fast, but not for the slow component (Figure 3B). In the elderly group, the *h*_max_ of the slow component of the raw signal, ^*s*^*h*_max_, was found increased (Figure 3C), while it did not change with the CBSI-treated signal. These changes were the opposite—similarly to Hurst exponent—regarding ^*f*^*h*_max_ in the young group (Figure 3D). Regarding the foci, for the raw HbT signals they were statistically the same in both age groups (Figure 3E), while for the fast component of the CBSI-enhanced HbT signal they were lower in elderly subjects (Figure 3F). Given that the (SD(HbO)/SD(HbR) ratios—the key element in CBSI model—did not differ between young and elderly groups (*p* = 0.543), the above significant differences should be regarded as real.

Age-related differences remained significant when the slow and fast components were compared. Specifically, component-wise contrast – defined as ln(^*s*^Ŝ_σ_(*N*))/ln(^*f*^Ŝ_σ_(*N*))—turned out to be significantly different between the age groups (*p* = 0.03). A concomitant—albeit non-significant—decrease characterizing the fast component (*p* = 0.405) for focus contributed to an overall increased ratio of foci (*p* = 0.067).

Comparing the multifractal parameters of cerebral hemodynamic fluctuations of female and male subjects, the only significant difference was observed for their HbO slow component. Incorporating age groups rendered the gender-related differences non-significant.

In order to prove that the significant differences in endpoint parameters seen in Figure 3 attribute to alterations in a single subject, endpoint parameters related to slow and fast component were statistically evaluated in combination as dependent variables in multivariate analysis. When scale-free endpoint parameters of the same kind [*Ĥ*(2), *h*_max_] were combined, MANOVA revealed a strong correlation (*r*^2^ > 0.7). Furthermore, significant multifractal endpoint parameters of slow component of raw HbT and fast component of CBSI HbT showed joint significance in a multivariate analysis (*p* = 0.045, Wilk's test) suggesting the coincident change of both components. Taken it together, these findings indicate that the observed alterations in the multifractal endpoint parameters resulted from subject-wise aging. As to the key geometrical parameters of the multifractal scaling functions, *p*-values obtained from Wilk's test indicated an overall non-significant influence of age. Of note the two main estimates of the analysis – *Ĥ*(2) and ln(Ŝ_σ_(*N*) – showed positive correlation for the fast component of the CBSI-pretreated (*r*^2^ = 0.46) and the slow component of the raw HbT signal (*r*^2^ = 0.58). The *p*-values of the statistical analyses are summarized Table 1.

**Table 1:** Significance of gender-related differences (*p*-values).

	**Parameter**	**HbO–raw**	**HbR–raw**	**HbT–raw**	**HbT–CBSI**	**HbO vs. HbR**
Slow component	*Ĥ*(2)	0.938	0.086	0.559	0.437	0.673
	*h*_max_	0.600	0.273	0.849	0.416	0.656
	Δ*H*_15_	0.828	0.671	0.305	0.591	0.852
	*fwhm*	0.823	0.731	0.456	0.581	0.861
	ln(*^*s*^Ŝ_σ_*(*N*))	0.408	0.539	0.273	0.296	0.494
Fast component	*Ĥ*(2)	0.845	0.442	0.503	0.205	0.378
	*h*_max_	0.115	0.060	0.140	0.141	0.049
	Δ*H*_15_	0.028	0.189	0.196	0.066	0.140
	*fwhm*	0.014	0.220	0.247	0.071	0.138
	ln(*^*f*^Ŝ_σ_*(*N*))	0.053	0.302	0.082	0.119	0.044

### Influence of age and gender on the HbO-HbR relationship

#### Scale-wise fractal cross-correlation

**Figure 4:**
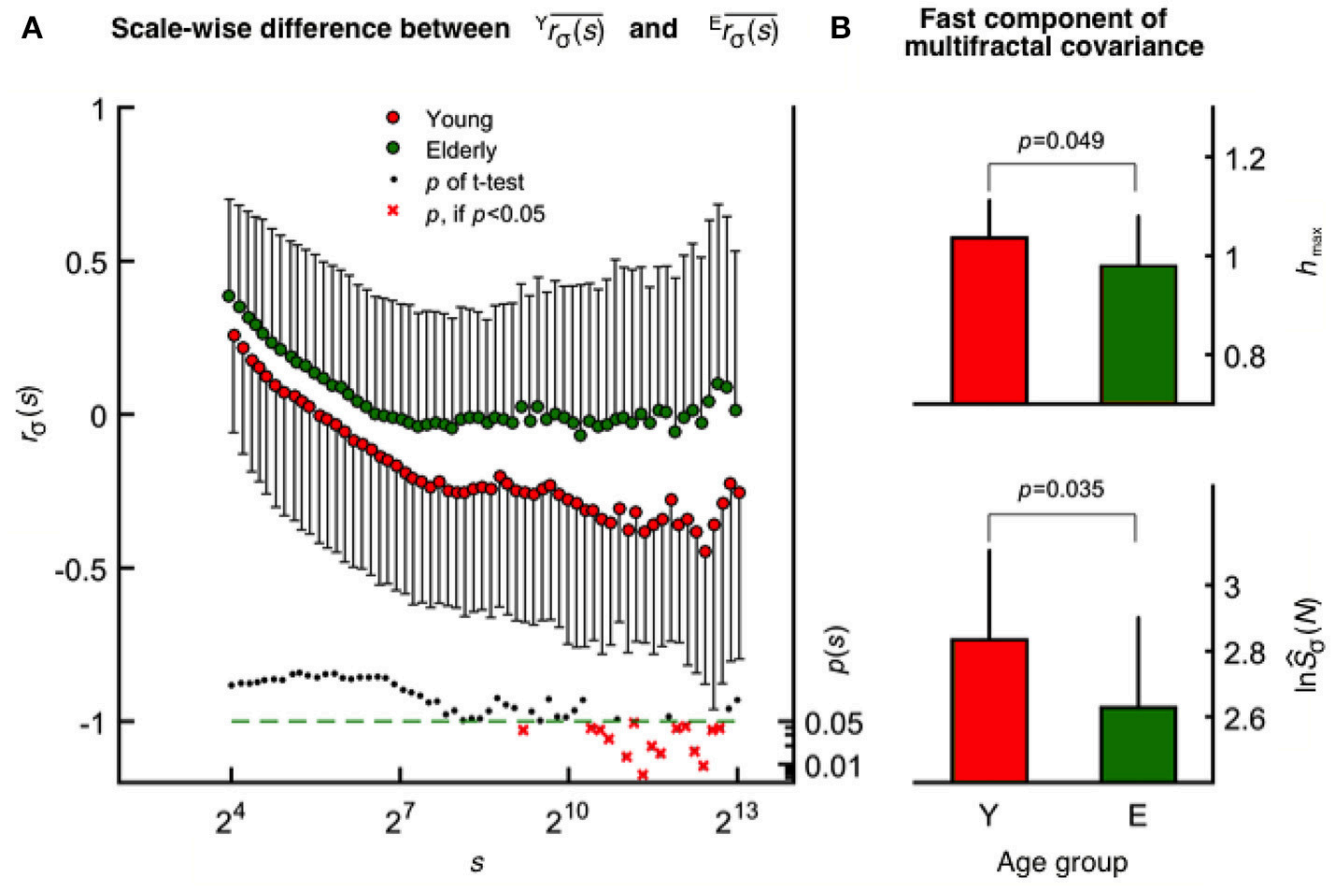
Age-related differences revealed by scale-wise and scale-free analysis of oxy- and deoxyhemoglobin relationship. **(A)** Fractal correlation coefficient of HbO and HbR as functions of scale in the elderly (upper) and young (middle) groups with the corresponding *p*-values (lower). At *s*_min_, these were above 0 and decreased gradually toward-1 as *s* approached *s*_max_ in both age groups. **(B)** Multifractal covariance of ^*f*^*h*_max_ (upper) and ln(^*f*^Ŝ_σ_(*N*); lower) of the fast component in the young and elderly groups.

The mean fractal scale-wise cross-correlation coefficient was found higher at all scales in the elderly group (Figure 4A). Importantly, this decrease in $\bar{r_{\sigma }\left(s\right)}$was more pronounced in young participants at higher values of *s* achieving significance (two-way ANOVA, confirmed by Tukey's *post-hoc* test) at a cluster of the corresponding high temporal scales (see the probability profile on Figure 4A). Based on this parameter calculated at these large scales, the oxy- and deoxyhemoglobin fluctuations are uncorrelated (random) among the elderly and were found anticorrelated in the young group. MANOVA revealed a statistical coincidence between the age-related increase in *r*_σ_(*s*) at specific high scales (corresponding to 2,172, 2,414, 2,683, and 2,982 s) and the same alteration observed for each multifractal endpoint parameters [^*s*^*h*_max_, ^*s*^*Ĥ*(2) of the raw HbT and ^*f*^*h*_max_, ^*f*^*Ĥ*(2) of CBSI-treated HbT]. In addition, concerning *r*_σ_(*s*) and ^T^*S*_σ_(*2, s*) as dependent variables their close relationship specifically appears at some of these aforementioned temporal scales. Since *r*_σ_(*s*) is determined by the dynamics of oxygen delivery and its extraction in the brain (i.e., supply and demand), these are key results for discussing the impact of aging on cerebral oxygenation.

#### Multifractal covariance

In contrast to scale-wise fractal correlation analysis when covariance is normalized by σ(*s*), the multifractal covariance analysis allows for a moment-wise characterization of the scaling properties of HbO-HbR coupling extending also for *q* ≠2. This method revealed a statistically significant age-related difference concerning the fast component [see ^*f*^*h*_max_ and ln(^*f*^Ŝ_*Cov*_(*N*)), Figure 4B], which is markedly correlated with ^*f*^*h*_max_ (*r*^2^ = 0.55) and ln(^*f*^Ŝ_*Cov*_(*N*)) (*r*^2^ = 0.69) obtained for CBSI-pretreated HbT signals across age groups.

Power-law scaling of the multifractal HbO-HbR covariance function may either originate from the independent scale-free variance profiles of HbO and HbR or as from the coupled fluctuations of the two. In case of the fast component, the significant contribution from the latter is confirmed for the whole study population, given that ^OR^*H*(*q*) derived from scale-wise covariances differed from (^O^*H*(*q*)+^R^*H*(*q*))/2 both obtained at *q* = 2 (*p* = 0.003, Wilcoxon matched pairs test). Moreover this pattern was absent in the elderly group (*p* = 0.116), but not in the young group (*p* = 0.006).

The correlation (*r*^2^) between dependent variables (i.e., multifractal endpoint parameters) captures the percentage of mutual variance of multifractal HbO-HbR covariance profiles and those obtained by SSC for the variance of CBSI-pretreated HbT signals. The percentage of mutual variance was the highest for ln(^*f*^Ŝ_*Cov*_(*N*)) (*r*^2^ = 0.81), and there was a strong relationship between their focus ratios (*r*^2^ = 0.72). However, the correlation was moderate for ^*f*^*h*_max_ (*r*^2^ = 0.56). To sum it up, results of multifractal covariance analysis seems rather consistent with an altered fast component of the CBSI-pretreated HbT signals.

### Significance of fractal scale-wise cross-correlation

**Figure 5:**
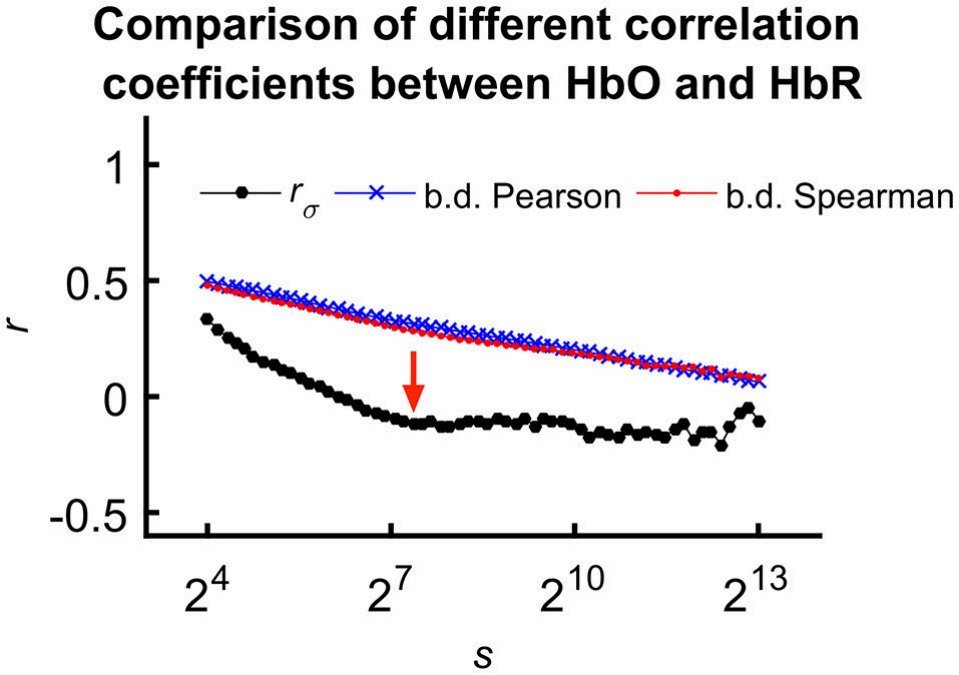
Comparing means of different correlation coefficients between HbO and HbR calculated for all subjects. We explain the less correlated values observed for *r*_σ_(*s*) with the effect of calculation order on trends in the signal. In case of the mean Pearson- and Spearman-coefficients, detrending and averaging step come after calculating the correlation effectively neglecting the fundamentally scale-free character of the processes. However, considering fractal scale-wise correlation coefficient, this latter step is the last preceded by averaging of bridge-detrended variances at a given scale. Please also note the characteristic transient—indicated by red arrow—only appearing in *r*_σ_(*s*).

For the sake of comparison, we calculated fractal scale-wise cross-correlation and averaged running Pearson and Spearman correlation coefficients at the same time scales (Figure 5).

**Figure 6:**
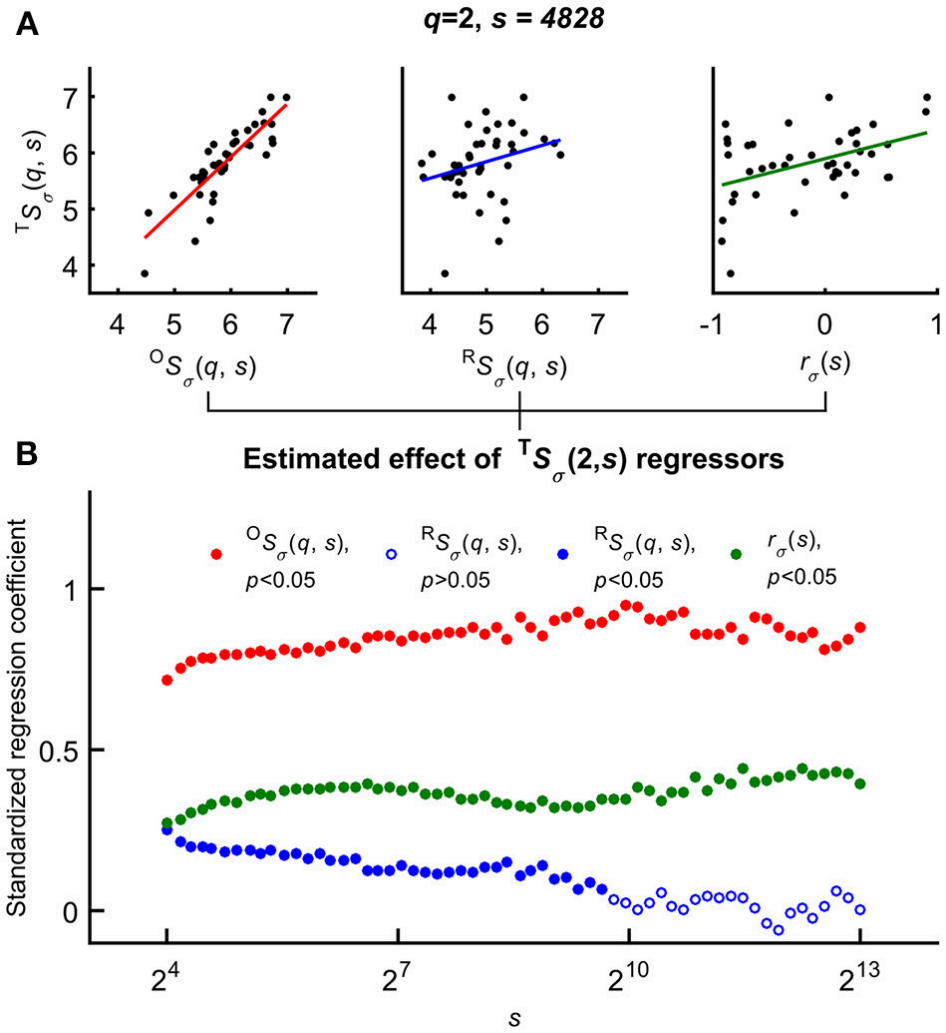
Significance of multiple regression tests. **(A)** Scaling function value of HbT as a function of ^O^*S*(*q, s*) (left), ^R^*S*(*q, s*) (middle) and scale-wise fractal cross-correlation coefficient (right) acquired at equal scale and moment (exemplary case at *q* = 2 and *s* = 4828). **(B)** Regression coefficients and their significance (closed circle for cases of *p* < 0.05) related to scaling function values of HbO, HbR; and *r*_σ_ as functions of scale *s*. The estimated effect were significant for all *s* values in case of ^O^*S*(*q* and *r*_σ_(*s*), and the lower standardized regression coefficients indicated the weak (not significant for all scales) effect of ^R^*S*(*q, s*). For further details, see main text.

Variability of the HbT scaling function profiles at *q* = 2 and for all scales was explained as related to the independent variables–*r*_σ_(*s*), ^O^*S*_σ_(2, *s*), and ^R^*S*_σ_(2, *s*)—based on the Bienaymé-formula given in Equation 2 (Figure 6 top). First, a series of multiple regression tests—not yet accounting for the effect of age and gender—were performed across all temporal scales that yielded positive correlation between each regressors and ^T^*S*_σ_(2, *s*). Importantly, the standardized regression coefficients proved to be significant for *r*_σ_(*s*) in case of all temporal scales. The highest estimated effects were observed in case of ^O^*S*_σ_(2, *s*) while this test revealed the weakest effect of ^R^*S*_σ_(2, *s*) being a non-significant regressor of ^T^*S*_σ_(2, *s*) at high scales (note the empty blue circles on Figure 6 bottom).

Subsequently gender and age were incorporated in the multiple regression model as categorical predictors to evaluate their influence on the *p*-value of correlation between the covariates and variance profiles related to CBV fluctuations (^T^*S*_σ_(2, *s*)). In the GLM framework the appropriate choice was AnCova or separate slopes model depending on the prerequisites of each approach. Age in of itself turned out to be not determinant of scale-wise hemodynamic fluctuations captured by ^T^*S*_σ_(2, *s*). It is the scale-wise cross-correlation on the basis of which the impact of aging could be explained (Table 2). These results confirm the outcome of multiple regression analysis showing a significant and strong relationship between ^T^*S*_σ_(2, *s*) and ^O^*S*_σ_(2, *s*) and a less steep but still significant relationship between the dependent variable and the fractal scale-wise cross-correlation coefficient.

**Table 2:** Homogeneity of Slopes Model/Separate Slopes Model/Covariance analysis results.

**Effect**	**Significance of the effect**
Gender or Age (*per se*)	Non-significant for any scale
Gender or Age (with interaction)	*Significant for 444 sec (Gender x Age x ^*O*^S_σ_ x ^*R*^S_σ_), Significant for 1758 sec (see below)*
^O^*S_σ_* (*per se*)	*Significant for all scales*
^R^*S_σ_* (*per se*)	*Significant for time scales between 8 and 400 sec*, non-significant for all time scales above 400 sec
^O^*S_σ_* or ^R^*S_σ_* (with interaction)	*Significant for 444 sec (see above), significant for 610 sec (^*O*^S_σ_ x ^*R*^S_σ_ x r_σ_), significant for 1758 sec (see below)*
*r_σ_* (*per se*)	Significant for all scales
*r_σ_* (with interaction)	*Significant for 444 sec (see above), significant for 1758 sec (for all interactions, with the exception of Gender x r_σ_)*

*Interaction effects are denoted as “x”*.

## Discussion

In this study, we found that hemodynamics in the human brain cortex captured by NIRS technology in most of the cases exhibited a bimodal multifractal scaling emerging from a range of low and high temporal scales (slow and fast components, respectively). We suggest relating the slow component of the raw HbT signal to dominantly vascular (vasogenic, i.e., non-neural) dynamics, while we consider the fast component primarily resulting from neurovascular coupling (i.e., neurogenic component). In order to demonstrate the impact of healthy aging, CBSI pretreatment of the raw HbT signal was necessary to enhance the neurovascular contribution in the fast component. Our main result is two-fold: first, we demonstrate that the vasogenic hemodynamics (CBV fluctuations proportional to HbT concentration changes) show increased long-range autocorrelation in the elderly group compared to the young group which is in agreement with what we had found previously applying monofractal analysis (^low^PSD_w,e_ method^5^) within comparable scaling ranges (Eke et al., 2006). Second, we show that the fluctuations of the neural component are less correlated in the elderly group. This opposite influence of healthy aging on the slow vasogenic and the fast neurogenic fluctuations is consistent with an attenuated NVC. In support of this notion, we evidence that age-dependent alterations in HbO-HbR relationship is a manifestation of altered neurovascular coupling and also a determinant of scaling properties of CBV dynamics. Specifically, in *in silico* experiments we substantiated that (i) a decreased correlation in neurogenic fluctuations is attributed to decreased incoming signaling, (ii) HbO-HbR relationship became more correlated due to aging either as a result of decreased incoming signaling concomitant to lesser hemodynamic response or from increased vascular stiffening. These alterations do indicate that linear CBV dynamics is susceptible to aging. In contrast, non-linear CBV dynamics is spared by aging as demonstrated in unaltered degree of multifractality.

Multifractality of cerebral hemodynamics has been investigated extensively in case of blood oxygen level dependent (BOLD) signals using functional magnetic resonance imaging (fMRI). The influence of brain activity was demonstrated in the pioneering work of Shimizu et al. (2004). Later, findings on the topology of multifractal parameters and methodological refinements were reported (Wink et al., 2008; Ciuciu et al., 2012). On data from a publicly available imaging repository (Biswal et al., 2010), the effect of age and gender on multifractal spectrum was shown for resting-state fMRI-BOLD signals (Ni et al., 2014). While rsNIRS signals have been made subject to multifractal analysis in an earlier feasibility study (Dzung, 2010; Quang Dang Khoa and Van Toi, 2012), our study—to the best of our knowledge—is the first reporting on it elucidating some of the key underlying mechanisms using a scrutinized dataset with proven true multifractality.

### Multifractal CBV dynamics

**Figure 7:**
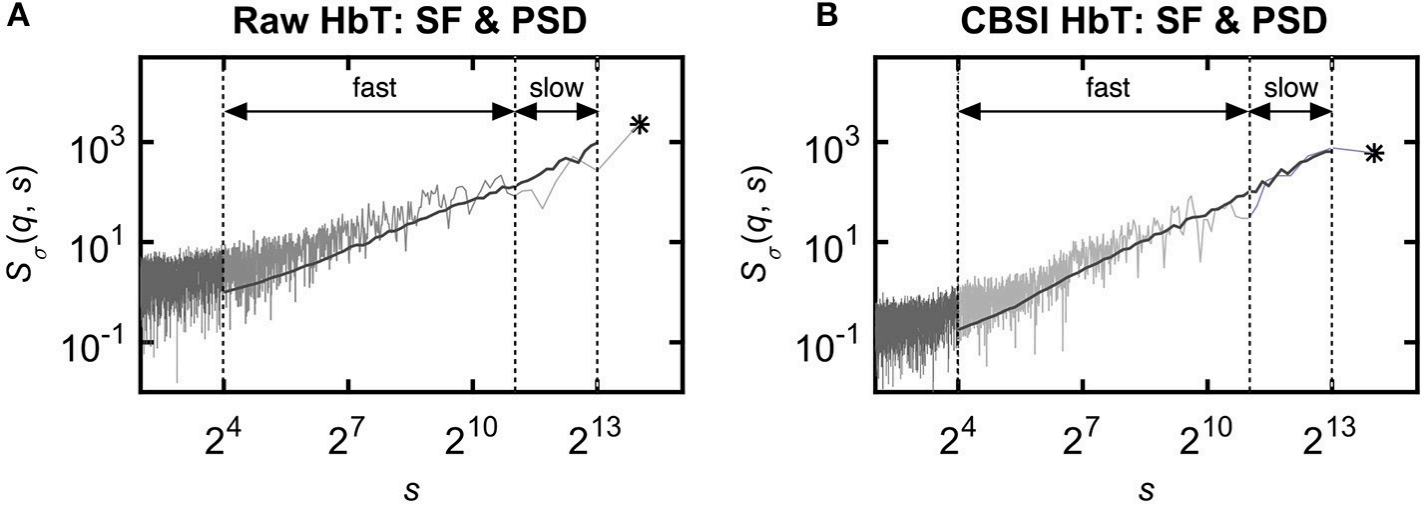
Relationship between power spectrum and scaling function profiles obtained by the FMF-SSC method. Although multifractal analyses were performed in the time domain, its design and results can be interpreted in the frequency domain as well owing to the explicit relationship between representation of dynamics in the temporal and the spectral domains both for the raw **(A)** and CBSI-pretreated **(B)** signal. The ordinate shows both frequency and temporal scale. It is the variance profile at *q* = 2 that corresponds well with the mean spectral estimates. The range of spectral estimates fall in between *S*(*q, s*) profiles for *q* = 15 and *q* = −15. The fractal scaling range emerges from the (non-fractal) noise (of biological origin) dominating the low temporal scales. It contains a breakpoint, which separates *S*(2, *s*) into a slow (associated with high temporal scales) and a fast (associated with intermediate temporal scales) scale-free component. This appears as a low- and a very-low frequency spectral band in the frequency domain. For further details, see text. Component-based focus is indicated by asterisk (^*^) as ln(Ŝ_σ_(*N*)) for the raw **(A)** and ln(Ŝ_σ_(*N*)) for the CBSI-pretreated HbT signal **(B)**.

CBV dynamics in the human brain cortex was shown to follow a complex, scale-free temporal pattern in the frequency domain that could be captured in the 1/*f* ^β^ model, where β is spectral index (Eke et al., 2006). The variance profile at *q* = 2 is analogous to the power spectrum, thus their apparent similarity can be readily shown (Figure 7). The estimated β obtained by ^low^PSD_w,e_ method is directly related to *Ĥ*(2), given the explicit relation of *H*(2) = (β+1)/2 (Eke et al., 2000). In fact, the power spectrum is equivalent to the Fourier transform of the signal's autocorrelation function according to Wiener-Khinchin theorem (He et al., 2010)^6^. Since its decay follows a power law with 2*Ĥ*(2)−1 as its exponent, *Ĥ*(2) and $\hat{\beta }$ are interpreted as equivalent measures of LRC in the fractional Gaussian noise / fractional Brownian motion framework (Eke et al., 2002).

It should be recalled that Fourier transform builds on independency of frequency components. As multifractality can emerge from interactions between multiple time scales (Ihlen and Vereijken, 2010), it cannot be captured in the power spectrum alone. Nevertheless, its presence still could be detected in the form of phase-amplitude coupling [nested frequency, see Ref. (He et al., 2010)]. Thus capturing multifractality in the time domain can reveal the underlying multiplicative interactions between the temporal scales of the observation with Δ*H*_15_ and *fwhm* as the measures of these cross-scale interactions (Ihlen and Vereijken, 2010).

#### Separation of neurogenic and vasogenic multifractal dynamics: CBSI-pretreatment

In pursuit of the physiological origin of hemodynamic fluctuations, the analyses were performed both on raw and CBSI-pretreated (Cui et al., 2010) data. In addition, we carried out *in silico* experiments to substantiate the need of this preprocessing step to identify components dominated by vasogenic and neurogenic influences, respectively (see Supplementary Material). CBSI method builds on the assumption that maximally correlated fluctuations of HbO and HbR are not related to neural activity. Given our recent demonstration of CBSI pretreatment enhancing the neuronal component in the signal (Racz et al., 2017) our two-teared approach of signal processing allows for a distinction between influences of neuronal and non-neuronal nature in this study; aspects of particular interest in the aging process. Accordingly, the age-related differences of the calculated multifractal measures revealed for the fast component of the pretreated signal should reflect altered neurogenic fluctuations. Vice versa, we found that the significant age-related differences in the multifractal indices obtained for the slow component of raw NIRS signals disappeared after applying CBSI. This indicated that the slow component was non-neural, referred to as vasogenic.

Some authors pointed out specific limitations of CBSI to isolate the neural component in the signal in fNIRS studies using various stimulus response paradigms (Scholkmann et al., 2014). Although the neural activity readily and always induces anticorrelated dynamics in HbO and HbR—as postulated in CBSI –, the response to a specific task may elicit systemic confounding effects, too. In this regard, CBSI cannot be considered to be immune to global effects (Tachtsidis et al., 2008). Nevertheless, as we present results based on the analysis of resting-state NIRS observations we do not need to deal with such confounding influences in task.

In principle, we could not *a priori* exclude that CBSI-pretreatment would not distort the signal. The formulation of the pretreatment algorithm allows for analytical considerations about the influence of CBSI on multifractal parameters, which in cases of other pretreatment algorithms would be more difficult to make. In particular, the above step in the algorithm can be shown to affect only the scale-dependent measures such as ln(Ŝ_σ_(*N*)) and breakpoint scales but not the scale-free parameters (*Ĥ*(2), *h*_max_, Δ*H*_15_, and *fwhm*), unlike with various filtering methods (Valencia et al., 2008). Nevertheless, these scale-dependent influences spared the impact of age on scale-free parameters because mean ^O^σ(*N*)/^R^σ(*N*) were found very similar in all measurement groups.

#### Origin of multifractality in resting-state hemodynamic fluctuations

Fluctuations of rsNIRS signal related to neural activity should be viewed as a sampled representation of an interim stage from intrinsic signal generation throughout the brain to the region of interest (ROI), where it is transformed into hemodynamic fluctuations. The regionally recorded rsNIRS signal—aside from systemic influences—is produced by the NVC driven by incoming signaling. Directly, it represents the hemodynamics within a population of vessels behaving like viscoelastic balloons in the ROI. As to the non-neural component of the rsNIRS signal, a likely origin of scale-free behavior is the numerous weekly coupled vascular source (diameter-dependent segmental oscillations along the arterial tree) blending into a fractally correlated pattern (Colantuoni et al., 1994).

Signal generation also raises questions about the spatial dynamics and resting-state functional connectivity. Regarding the incoming neural activity, electrocorticography records captured across various locations in the brain cortex have been shown scale-free temporal structuring (He et al., 2010; He, 2011). Moreover, the power spectral density of cortical EEG exhibits scale-free structuring not only in the temporal but—in an interrelated manner—in the spatial domain, too, (Freeman et al., 2003). Specifically, as demonstrated by these authors, fluctuations spanning from high-frequency/low-power bands to the low-frequency/high-power ranges reflect upon neural events propagating across the micro-meso-macro scales representing contributions from ion channels, across gyri all the way to those of lobes, respectively. Hence a PSD and scaling function representations of neurodynamics and coupled hemodynamics could be viewed as capturing the information flow within the system from its sources via inhomogeneous network routes eventually converging onto the signaling input of the monitored ROI (Buzsaki, 2006). The sampled representation of this process will typically show inhomogeneously distributed fluctuations, visible as intermittent periods of small and large variability; genuine properties of multifractal processes (Ihlen and Vereijken, 2010).

When the multiplicative cascading process was extended into the spatial domain, a description was obtained comparable to the one by the self-organizing branching process (Zapperi et al., 1995); a refined extension of the SOC model (Bak et al., 1987). The inference is that the intermittent in essence multifractal temporal patterns and the inhomogeneous incoming network connections are manifestations of the same phenomenon: emergence of intermittent regional activity from multiple sites of the brain converging via multiplicative interactions between spatiotemporal scales as integrated incoming signaling in the ROI.

Indeed, our findings related to fractal dynamics could potentially reflect the presence of SOC (Bak et al., 1987) in the observed physiological subsystems shaping cerebral hemodynamics in the ROI. SOC, substantiating the 1/*f* noise-type neurodynamics of the human brain, builds on the notion that the brain dissipates the local low-frequency perturbation elicited by external or internal stimuli without any particular spatial or temporal scales (Stam, 2005; Bullmore et al., 2009; Chialvo, 2010; Sporns, 2011). When interactions between scales occur, multifractality can readily emerge in systems showing properties of SOC (Tebaldi et al., 1999; Lima et al., 2017). Of relevance, in a recent rsNIRS study using 16 channels sampling of resting-state hemodynamics in the PFC, it has been evidenced that the presence of critical state in resting-state dynamic functional connectivity (Racz et al., 2018). In sum, the measured signals are considered as a composite of hemodynamic fluctuations of vasogenic and those elicited by incoming signaling of neurogenic origins with NVC as the link between the two.

#### Interpretation of multifractal endpoint parameters

The vasogenic component of the rsNIRS signal in terms of its observed multifractal temporal patterns can be interpreted as a consequence of attenuated neurovascular coupling—reflected by an anticorrelated → random shift in the fractal cross-correlation of HbO and HbR. As to the neurogenic fluctuations here we explain the altered multifractal endpoint parameters resulting from interactions between multiple time scales along functional connections (Ihlen and Vereijken, 2010). Along with our focus-based multifractal formalism (Mukli et al., 2015) and a small world implementation (Watts and Strogatz, 1998) of the concept of self-organized criticality (Mandelbrot, 1974; Ihlen and Vereijken, 2010) offer a concise framework for the interpretation of the results obtained in this study the way outlined in the followings. Further details are provided below and please also see the Supplementary Material.

##### Linear dynamics: H(2) vs. h_max_

Since the degree of global LRC is quantified by *H*(2), its changes can be interpreted as increased or decreased persistence, meaning correlation of a non-stationary process (Eke et al., 2000; Herman et al., 2011). Furthermore, Deligniéres et al. established a relationship between network *degeneracy*—meaning partial overlap in heterogeneous functional connections—and output signal correlation (Delignières and Marmelat, 2013). Accordingly, the Hurst exponent does not only reflect upon global scale-free properties emerging from a network, but its degree of degeneracy, too.

While LRC—and thus *H*(2)—reflects global scale-free properties, the Hölder trajectory is a local scale-free measure varying along the signal. Although multifractal analysis of physiological data usually shows tightly correlating changes of *h*_max_ and *Ĥ*(2), the interpretation of *h*_max_ as a measure of correlation within the signal is only approximate, since it is associated with *q* = 0, not *q* = 2.

##### Non-linear dynamics: Δh_15_ and fwhm

Though Δ*H*_15_ is defined on *H*(*q*) and *fwhm* is derived from *D*(*h*), their excellent correlation – owing to the deterministic formalism established by Equations 3–5 – offers a rationale to interpret them together. The applications of *q*-order statistics reveals non-linear properties in scaling of the signal (Ashkenazy et al., 2003). Thus these two multifractal endpoints are indeed equivalent measures of multiplicative interaction between temporal scales of the observed dynamics process (Ihlen and Vereijken, 2010). Importantly, Δ*H*_15_ and *fwhm* should be regarded as indicators of non-linear dynamics (Gómez-Extremera et al., 2016; Bernaola-Galván et al., 2017).

Asymmetry of *D*(*h*)—an occasionally observed phenomenon—could be incorporated in the analysis of multifractality in terms of *W* = *W*+/*W*– where *fwhm* is equal to the sum of *W*+ and *W*– (Wink et al., 2008), corresponding to the width of left and right-half of the singularity spectrum. We calculated the *W* and found the shape of our singularity spectra symmetric and not affected by age and gender. We stress that testing for true multifractality based on Δ*H*_15_ statistics (for details see Appendix in Supplementary Material) is an essential prerequisite when it comes to evaluating changes in these endpoint parameters in regard of multifractal CBV dynamics.

##### A scale-dependent measure of hemodynamic power: focus

The focus of the scaling function is a key element of our regression scheme for obtaining *H*(*q*) thus securing a robust estimate of *D*(*h*) free of inversion (Mukli et al., 2015). Importantly, it is also a robust scale-dependent statistics estimated at signal length as a point of convergence for the scaling function profiles. Since our analysis is based on the SSC method using bridge-detrended variance, Ŝ_σ_(*N*) is essentially the variance associated with the whole signal. Given that the coefficient of variance for our rsNIRS signals were the same, Ŝ_σ_(*N*) is also the measure of the signal mean, which is consistent with our SOC-simulations (Figure S3). In the frequency domain, it is analogous with the power of the DC-component of the signal (see the behavior of spectra and scaling functions on Figure 7 as *f* → 0).

Since its value is influenced by numerous other variables, conclusions regarding hemodynamic alterations can be drawn if focus is interpreted together with *Ĥ*(2). Given their analogous frequency domain parameter the hemodynamic power corresponding to a spectral band can be estimated. The separated components of the rsNIRS signal have distinct scaling ranges, and the area under the scaling function corresponding to such SRs is an approximation of summed logarithmic variance of the given component. This area can be explicitly calculated as it is proportional with ln(Ŝ_σ_(*N*)) and width of SR, and it also increases with decreased *Ĥ*(2). Given the straightforward relationship between summed variance and total hemodynamic power in a given temporal/frequency range, the obtained results for the area should be viewed as power of hemodynamic fluctuations associated with the isolated components.

#### The inference of bimodality

The majority of the measured rsNIRS signal showed bimodal scaling that was statistically confirmed by comparing the errors of fits for the bimodal and unimodal models. We used a scaling-range adaptive method to assess scaling exponents and multifractal endpoint parameters of the two components, which approach has already been used in our previous study (Nagy et al., 2017) and in other studies as well (Ge and Leung, 2012; Kuznetsov et al., 2013). The apparent structural heterogeneity in the scaling functions of our dataset (convex and/or concave transient range) prompted us to choose the robust moment-wise SR-adaptive method instead of an alternative (decomposition of scaling function) that was specifically designed to assess additive properties of bimodal scaling.

The bimodal analysis is an adaptive tool that separates two fractal SRs within an overall range of scales between the lowest temporal scale of 8 seconds (*s*_min_ = 16) and 4096 seconds (*s*_max_ = 8192). Although the Fourier transform does not assume an exact relationship between temporal scales and frequencies, we can still assign the non-fractal “noise” component below *s*_min_ to a spectral range from 0.125 to 1 Hz (i.e., Nyquist frequency). It is known that this band is dominated by fluctuations of systemic origin such as cardiac pulsation and respiration (Tian et al., 2009; Sassaroli et al., 2012), which is corroboratory regarding the exclusion of time scales shorter than 8 s.

Earlier we introduced the slow and fast components as dominantly neurogenic and vasogenic, in the followings the reader is taken through arguments substantiating this division. The fast and slow components were identified in the time domain within their respective analytical scaling ranges in the time domain. In a frequency domain representation, they correspond to the low- and very low-frequency oscillations (LFO and VLFO, respectively; Figure 7). The LFO/VLFO classification was used by authors evaluating fundamental spectral aspects of NIRS dynamics (Obrig et al., 2000; Schroeter et al., 2004; Li et al., 2013; Vermeij et al., 2014). LFO is generally regarded of neural origin and thus is a commonly investigated in NIRS studies of cognition (Chance et al., 1993) and functional connectivity (Sasai et al., 2011). Also, it substantiated the concept of fMRI-based functional connectivity ever since Biswal et al. (1995) analyzed cross-correlations in paired signals by clipping their power spectra to zero above 0.1 Hz in order to identify temporal coincidences in local activities thus excluding the global influences. It seems reasonable to separate the VLFO component since its dynamics is dominated by non-neural (Schroeter et al., 2004; Li et al., 2013; Vermeij et al., 2014), particularly endothelium-related mechanisms (Li et al., 2013; Chen et al., 2014). VLFO would manifest as the slow, vasogenic component in our study within the breakpoints and *s*_max_, while the range down to *s*_min_ corresponds to LFO (Figure 7). Nevertheless, the debate is still ongoing over further contributors to CBV dynamics such as vasomotion (Elwell et al., 1999) and Mayer-waves (Sassaroli et al., 2012); effectively being excluded from our analyses by setting *s*_min_ to 8 s corresponding to 0.125 Hz. In fact, vasomotion may show up, but within a narrow range of scales and at low fluctuation amplitudes thus having a weak effect if any on our scaling analysis. Please note that the assessment of bimodality and scale-free properties of both LFO and VLFO were only possible because the analyses were performed with a high-enough *s*_max_ as recommended by Nagy et al. (2017). The consequent statistical instability is compensated by our focus-based regression model.

### Healthy aging is associated with altered complexity of cerebral hemodynamics

In the present study, decreased *Ĥ*(2), *h*_max_, and ln(Ŝ_σ_(*N*)) were seen in the fast—neurogenic—component of the CBSI-pretreated NIRS signals of the elderly participants. In addition, we found an increased *Ĥ*(2) and *h*_max_ in the elderly group for the slow—vasogenic—component of the raw rsNIRS signal (Figure 3) with spared ln(Ŝ_σ_(*N*)) and multifractality (i.e., no difference in Δ*H*_15_ and *fwhm*). These changes in the multifractal endpoint parameters are consistent with attenuated neurovascular coupling concomitant to declining incoming signaling and impaired vascular responses.

#### Altered neurogenic component due to declining neurodynamics

Multifractal measures of the fast component (~ LFO) of the CBSI-pretreated NIRS signal revealed a difference between young and elderly subjects. Specifically, *Ĥ*(2), *h*_max_, and ln(^*f*^Ŝ_σ_(*N*))—a key parameter characterizing the overall decline in the neurogenic component—was found decreased among the elderly participants. In principle, a decrease of *Ĥ*(2) and *h*_max_ could have resulted from the contribution of biological noise. The decreased ln(^*f*^Ŝ_σ_(*N*)) can be directly interpreted as a decreased power in LF oscillations which was concluded by other studies, too (Schroeter et al., 2004; Li et al., 2013; Vermeij et al., 2014). Although the decline in power appears at all frequencies, a disproportionate decrease could still manifest from biological noise present across the lower frequencies first seen in our previous study (Eke et al., 2006). A recent numerical study clearly showed that varying signal/noise ratio by adding white noise yielded underestimation of *H*(*q*) (Ludescher et al., 2011). Hence our decreased *Ĥ*(*q*) and *h*_max_ of the fast component can at least in part be explained by the relative impact of biological noise progressively dominating the higher temporal scales. Although our choice of *s*_min_ exceeds this range of temporal scales, this factor must be taken into account in our interpretation of the observed alterations of the neurogenic component associated with the LFO range in our rsNIRS signals. In spite of the spurious estimates of *Ĥ*(*q*) attributable to the increased relative impact of biological noise, the conclusion of an overall decline in the neurogenic component can be still held. Nevertheless, please note that CBSI-pretreatment of raw HbT signal effectively removes the impact of non-anticorrelated dynamics in the signal. Therefore the impact of biological noise is unlikely and the observed changes should be regarded as real.

This allows us to interpret the decreased focus as a sign of decreased incoming signaling in line with the dominantly neurogenic origin of the LFO and the aforementioned *in silico* observations (Supplementary Material). As multifractality can be viewed as resulting from cross-scale spatiotemporal interactions (Monto, 2012), unchanged Δ*H*_15_ and *fwhm* suggest that healthy aging spared these interactions within the incoming networks.

As to the multiple mechanisms involved in neurogenic aspects of the aging process, based on our results we argue that even healthy aging leads to progressive attenuation in incoming signaling that to some unknown extent could be masked by impaired neurovascular coupling. The aging process is known leading to gray matter atrophy associated with dropping neuronal count (McGeer et al., 1984) and lower gray matter density (Sowell et al., 2003) along with impaired synaptic activity. The latter is known to be prevailing in postmenopausal women due to lower levels of estrogen (Gibbs and Aggarwal, 1998; Khan et al., 2013). In addition, an age-related decrease in the hemodynamic response elicited by cognitive task has been observed in the human prefrontal cortex by NIRS (Schroeter et al., 2004). Several studies have evidenced that significant changes in resting state functional connectivity take place in the aging brain at large and small spatial scales alike with inference to temporal dynamics (Ferreira and Busatto, 2013). For example, the structural changes occurring in the aging brain imply changes in its functional connectivity readily manifesting in altered parameters of complexity parameters (Sun et al., 2012). Specifically, global deleterious effects of older age has been reported on functional networks (Achard and Bullmore, 2007) mounting to “topological marginalization” like that of the prefrontal cortex due to segregation in the global network. It was suggested that even healthy aging disrupts the underlying networks by severing the long-range connections, especially in higher-function areas like the prefrontal cortex (Ferreira and Busatto, 2013). In a sample of subjects (19–80 years) a linear effect of age associated with impaired resting-state functional connectivity has been demonstrated (Mevel et al., 2013).

Indeed, when eliminating the multiplicative neural interactions across spatial scales in an interaction-dominant model of human cognition (Ihlen and Vereijken, 2010), more aggregated, focal activities remain. Putatively, this model behavior (global → focal shift in intrinsic activities of the brain) is capable of explaining the loss of complexity in our rsNIRS signal as the sign of decreased incoming signaling. Nevertheless, this takes place in the PFC during healthy aging to such an extent that would still not interfere with the cross-scale spatiotemporal interactions in the observed dynamics captured in unaltered parameters of multifractality.

A further theory suggests the role of dedifferentiation in the aging of physiological subsystems like the brain function (Sleimen-Malkoun et al., 2014). Experiment using a motor task paradigm indeed demonstrated that in addition to stronger activation in dedicated regions to a motor task, older adults generally exhibit activation of additional areas of the brain not or only marginally involved in young participants (Sleimen-Malkoun et al., 2014). Approaches relying on fMRI-based connectivity studies accounting for the difference in task and connectivity paradigms demonstrated that higher levels of activity coexists with disrupted connectivity (Sala-Llonch et al., 2015).

#### Altered vasogenic component due to impaired vascular responses

**Figure 8:**
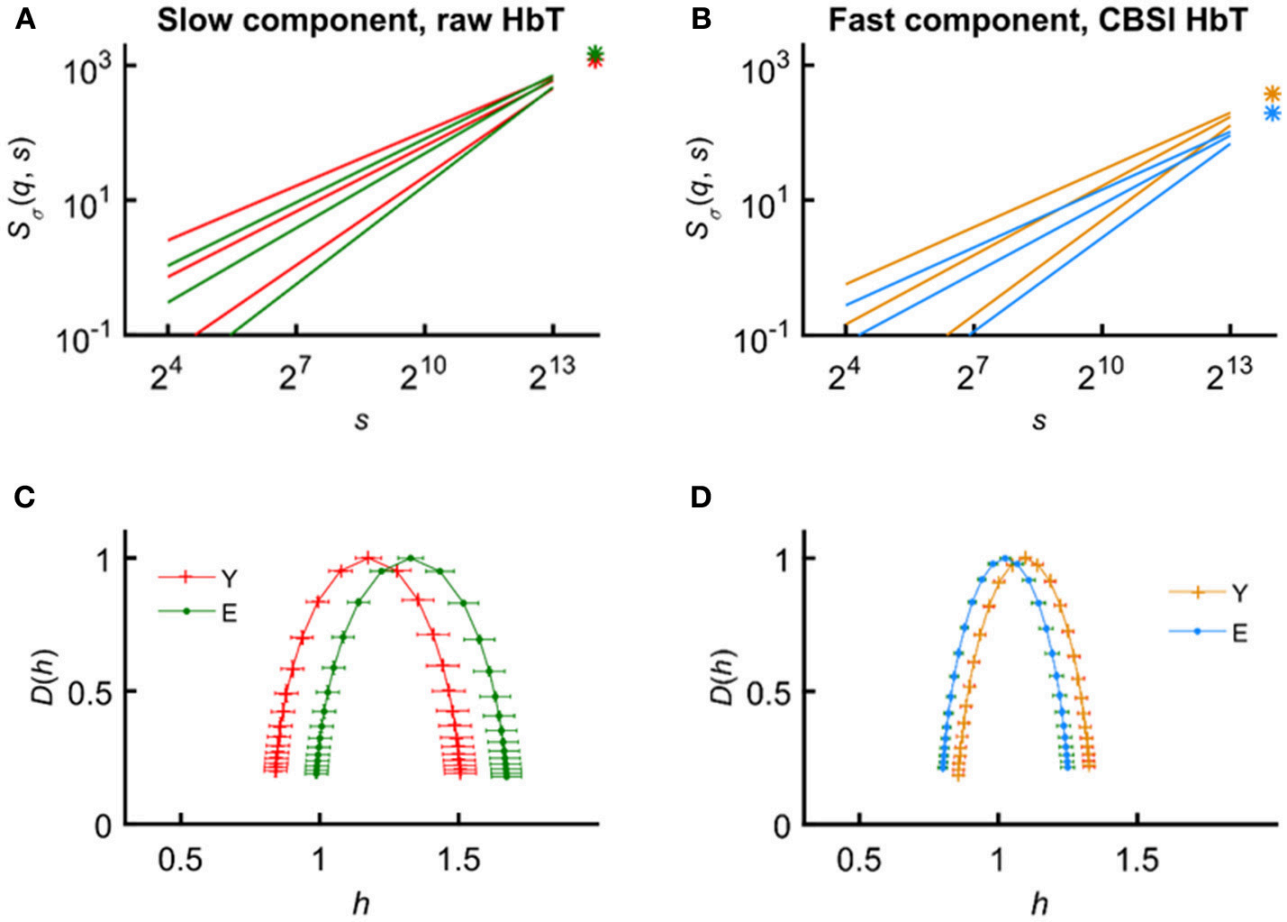
Input and output parameters of the focus-based multifractal formalism in the young and elderly groups for the slow (vasogenic) and fast (neurogenic) components of CBV fluctuations. Average scaling functions **(A,B)** and average singularity spectra **(C,D)** obtained for the raw and CBSI-pretreated HbT signal. Age coding: young—red **(A,C)**, blue **(C,D)**; elderly—green **(A,C)**, orange **(B,D)**. Preserved focus and increased correlation characterize the age-related changes of the vasogenic component **(A)**. Conversely, these parameters of the neurogenic component indicate a decline apparent in the average scaling functions **(B)**. The alteration of *h*_*max*_ is a good indicator of the right-ward shift of *D(h)* for the vasogenic **(C)** and a left-ward shift for the neurogenic component **(D)**. It also reveals altered correlation properties if the shape and distribution of singular behavior is not much affected, which happens to be the case in this study as the distribution of Hölder exponents was found symmetrical around *h*_*max*_ irrespective of age and gender.

The observations regarding the vasogenic component are compatible with those of our previous monofractal study using raw rsNIRS signals reporting on an increased spectral index β in the elderly suggesting the impact of age-related vascular sclerosis on CBV dynamics (Eke et al., 2006). The increased *Ĥ*(2) and focus indicate a more correlated vascular dynamics with decreased hemodynamic power in the elderly group. The mean singularity spectrum for the vasogenic component was found shifted to the right (reflected by *h*_max_) with maintained shape and width (Figure 8). Consistent with this view, we hypothesize that healthy aging leads to this increased correlation pattern in the vasogenic (VLF) component as a result of vessel stiffening and a decline in the endothelium-mediated (metabolic) regulation of cerebrovascular smooth muscle tone. Our evidence to this hypothesis is indirect, though: the result of CBSI-pretreatment and the temporal scales characterizing VLF hemodynamics. Nevertheless, this interpretation of our results is in agreement with several studies on this component of cerebral hemodynamics. In an rsNIRS study investigating the effect of age, Li et al. demonstrated (Li et al., 2013) decreased average amplitudes of spontaneous oscillations in the elderly. The latter authors assumed that the oscillations in the 0.005–0.02 Hz range originated from the endothelium. Aging has also been shown decreasing the responsiveness of distal segments of the arterial tree due to endothelial dysfunction (Toda, 2012) and increasing wall stiffness (Schroeter et al., 2004; Zhu et al., 2011; Wardlaw et al., 2013; Vermeij et al., 2014). The functional hyperemia studied by selectively interrupting endothelial signaling in the somatosensory cortex of rats confirmed its key role in mediating the very slow—maintained—hemodynamic response brought about by NVC (Chen et al., 2014). The vascular decline in the elderly thus may attenuate the local hemodynamic response, too.

As to the attenuation in local vascular dynamics, indeed, the local hemodynamic response elicited by *incoming* neural activity is known to be driven *locally* by fast glutamate-mediated signaling, and more *globally* by amine- and ACh-mediated neural systems (Attwell and Iadecola, 2002). Both have been shown declining with age (McGeer et al., 1984; Gibbs and Aggarwal, 1998; Attwell and Iadecola, 2002). The above scenario may indicate that an impaired neurovascular coupling (Fabiani et al., 2014; Tarantini et al., 2017) could to some degree mask the effects of deterioration of connectivity on neurogenic signal complexity.

### Implications of HbO-HbR relationship

Age-related physiological dysregulation is essentially a gradual and typically irreversible loss of regulatory control originating from structural instabilities in regulatory systems (Cohen, 2016). We shall discuss this phenomenon concerning parameters reflecting neurovascular coupling with coupled HbO-HbR dynamics in its focus. We used multifaceted approach for its characterization: (i) scale-wise fractal cross-correlation, (ii) multifractal covariance and (iii) statistical analysis of Bienaymé-formulation of the HbO-HbR relationship. At this end, we interpret our results as the impact of age on the output of an integrated system of neurodynamics, coupled HbO-HbR dynamics and hemodynamics as genuinely interrelated aspects of neurovascular coupling.

#### Age-related increase in scale-wise fractal cross-correlation

In elderly participants, the higher *r*_σ_(*s*) capturing HbO-HbR relationship indicate the relatively larger contribution of correlating systemic hemodynamics to the recorded NIRS signal (which) is also consistent with declining neurodynamics. Furthermore, scale-wise correlation coefficients were found significantly elevated for rather high temporal scales directly influencing this domain of the variance profiles associated with the vasogenic component (Figure 4A). In a certain range of scales characterizing VLFO, the increase in *r*_σ_(*s*) values were significantly associated with the ^*s*^*h*_max_ and ^s^*Ĥ*(2) of the raw HbT. Given the demonstrated association between endothelial-mediated responses and VLFO (Stefanovska et al., 1999), the pattern found in our study should be regarded as evidence supporting the endothelial contribution to the age-related increase in ^*s*^*h*_max_ and ^s^*Ĥ*(2).

Analyzing the coupled fluctuations of oxy- and deoxyhemoglobin has been used to assess cerebral oxygenation changes and the underlying processes (Reinhard et al., 2006; Wylie et al., 2009; Pierro et al., 2012). There is scarce evidence to determine the effects of aging on their relationship and the rationale behind the utilized parameters is also debatable. Please note the difference in the scale-wise pattern of *r*_σ_(*s*) only showing a clear gradual decrease below ≈50–100 s (see the red arrow on Figure 5). It is reasonable to assume that such pattern reflects the relative contribution of mechanisms eliciting correlated or anticorrelated chromophore dynamics. Supposing the origin of the anticorrelated dynamics within the local balloons, and regarding the correlated dynamics mainly of systemic origin, this pattern may be informative of a relative impact of local and systemic hemodynamics on our measured rsNIRS signals. Specifically, local determinants of oxygen supply and extraction dominate the correlation across wide range of frequencies below 0.01 Hz (Stefanovska et al., 1999) emphasizing the potential contribution of spatiotemporally sustained response mediated by astrocyte-endothelial signaling (Iadecola and Nedergaard, 2007).

#### The significance of non-linear relationship revealed by multifractal covariance analysis

While scale-wise cross-correlation analysis does not reveal scale-free properties, multifractal covariance analysis is specifically designed to characterize LRCs and multifractality in the coupled HbO-HbR dynamics. The covarying fluctuations of HbO and HbR originate from their respective individual fluctuations and the directly coupled dynamics of the exchange between the two compartments. Following the approach of Kristoufek (2011) the deviation of ^OR^*Ĥ*(2) from (^O^*Ĥ*(2)+^R^*Ĥ*(2))/2 indicates oxygen exchange within the hemoglobin pool. We found a clearly significant decrease of ln(^*f*^Ŝ_*Cov*_(*N*)) in the elderly (Figure 4B) indicating weakening of the coupled fluctuation of HbO and HbR. Indeed, the inference is an upset balance between oxygen demand and supply. Given the strong association between the foci of the fast component of the covariance scaling function and of CBSI-pretreated signal scaling function, this change supposedly can be attributed to the age-related decline in neurodynamics. Although at a weaker significance and lower correlation with the corresponding multifractal endpoint parameter (obtained for multifractal covariance analysis and multifractal analysis of CBSI-pretreated signals, respectively), the decreased ^*f*^*h*_max_ of HbO-HbR covariance in the elderly participants besides its similar pattern with ^*f*^*h*_max_ of neurogenic fluctuations supports this notion. Clearly, more evidence is needed coming from measurements and synthesized datasets to elaborate and consolidate physiological models for this interpretation.

Although our approach is essentially similar to the multifractal detrended- (Zhou, 2008), height cross-correlation (Kristoufek, 2011) analyses and cross-wavelet analysis (Ciuciu et al., 2014; Jiang et al., 2016), this is the first paper describing and utilizing the bivariate adaptation of multifractal SSC analysis. Owing to the similarities between our implementation and other time domain algorithms a reliable parameter estimation was expected. A performance characterization of multifractal covariance analysis on synthetic signals with known degree of correlation, while is certainly desirable, but is beyond the scope of the present study.

#### The importance of coupled HbO-HbR fluctuations driving cerebral hemodynamics

Physiologically, oxygen consumption related to neural activity is not blood flow-limited, but rather neural activity controls the CMRO_2_ and cerebral hemodynamics (Raichle and Mintun, 2006). Consequently, it is plausible to explain variations of CBV fluctuations (proportional to the measured HbT signal) as a function of HbO-HbR relationship providing insight in variations in cerebral oxygenation. The explicit deterministic, quantitative relationship between key variables of the study established by Bienaymé-formula (Equation 2) offers an explicit way assigned by GLM to evaluate their role with special emphasis on *r*_σ_(*s*).

Scale-wise cross-correlation is a robustly significant determinant of HbT fluctuations at all temporal scales within the framework of GLM. Omitting either age and gender from the statistical analyses (i.e., multiple regression, Figure 6) or incorporating these categorical variables in it (AnCova/separate slopes) yields essentially the same outcome on the role of *r*_σ_(*s*). Considering the impact of age on *r*_σ_(*s*) and the age-corrected dependence of *r*_σ_(*s*) CBV fluctuations (see Table 2) it can be concluded that age exerts its effect on ^T^*S*_σ_(2, *s*) in part via *r*_σ_(*s*) shifting toward uncorrelated chromophore dynamics. The impact of weakened HbO-HbR coupling in the elderly—supposedly due to declining neurodynamics—is clearly seen in the age-related changes of multifractal endpoint parameters characterizing cerebral hemodynamics.

### Limitations and future perspectives

Acquiring additional modalities would have allowed for a more straightforward physiological interpretation of the signal. Specifically, transcranial Doppler measurement (capturing blood flow velocity in the middle cerebral artery) or continuous blood pressure records offer measures on systemic influences that could have been regressed out. To compensate, we applied CBSI to remove these influences from the signal. Further development and testing of robust pre-processing methods are in place to further enhance the interpretation of multifractal measures of physiological processes.

The intimate relationship between multifractal hemodynamic fluctuations and functional connectivity has been demonstrated and characterized on intrinsic fMRI networks (Ciuciu et al., 2014) and by revealing scale-free network dynamics in the prefrontal cortex captured by NIRS (Racz et al., 2018). In this comparison, our single-region measurement measurement appears like a limitation (Novi et al., 2016). However, in fact, we present an analytical framework which is capable of integrating aspects on incoming signaling with those of regional hemodynamics elicited by neurovascular coupling.

The tools developed for characterizing the coupled HbO-HbR dynamics have potentials in other applications, too. The fractal scale-wise correlation analysis captures linear aspects while the multifractal covariance analysis adds a non-linear dimension to the assessment of coupling between non-stationary time series. These methods open new ways in uni- and multimodal applications to investigate functional connectivity or neurovascular coupling, respectively.

## Conclusions

Mono- and multifractal approaches have greatly enriched our insight of biological complexity in particular that of the brain (Bullmore and Sporns, 2009; Herman et al., 2009; Ihlen and Vereijken, 2010; Nagy et al., 2017; Racz et al., 2018). To the best of our knowledge, this study is the first using consolidated datasets with tested and proven correlation-type multifractality for an in-depth characterization of resting-state NIRS fluctuations. Here we interpret the multifractality of single-region cerebral hemodynamics as resulting from neurogenic oscillations via cross-scale interactions blending into a scale-free intermittent arrhythmic pattern via neurovascular coupling. However, the intrinsic and endothelium-evoked heterogeneous oscillations of the vascular smooth muscle tone give rise to multifractality of vasogenic fluctuations. The multifractal endpoint parameters obtained from raw and pre-processed signal attest to the impact of healthy aging on cerebral hemodynamic fluctuations in the human prefrontal cortex. Specifically, we report that the total power of very-low frequency—vasogenic—oscillations of CBV decreased due to a preserved value of focus and an increase of LRC, (^*s*^*Ĥ*(2)); the latter is concomitant to the right-shifting singularity spectra [*D*(*h*) along with its ^*s*^*h*_max_]. As to the hemodynamic fluctuations elicited by neural activity—related to low-frequency oscillations—we show a general decline indicated by decreased *Ĥ*(2), *h*_max_, and focus of the neurogenic component. On the contrary, parameters reflecting degree of multifractality are the same in the group of young and elderly subjects which demonstrates non-pathological aging spares non-linear hemodynamics. In case of the elderly participants, the anticorrelation of HbO and HbR fluctuations were barely present at high temporal scales, while an attenuated cross-correlation was revealed by multifractal covariance analysis. We show that the impact of age on the parameters of neuro- and vasogenic components must have resulted from the age-related alterations in HbO-HbR coupling. In our study, the HbO-HbR relationship appears as a key element directly influenced by the neuronal activity and directly coupled to CBV dynamics via neurovascular coupling which seems like sensitive to aging. We suggest that decreased incoming signaling and the prevalence of an altered pattern of moment-to-moment HbO-HbR coupling could contribute to the mismatch between oxygen demand and supply. Together with vascular dysfunction could well be considered as factors behind the observed age-dependent alterations of cerebral hemodynamics.

## Author contributions

PM adapted the multifractal analytical framework to resting-state NIRS signals, performed the analysis on measured data and wrote the manuscript. ZN developed methods and helped writing the manuscript. FR implemented a testing framework and assessed true multifractality of the measured data. PH carried out the measurements. AE helped developing and writing the manuscript and provided conceptual guidance in the study.

### Conflict of interest statement

The authors declare that the research was conducted in the absence of any commercial or financial relationships that could be construed as a potential conflict of interest.

## Footnotes

^1^*q* takes all integer values in this interval.

^2^Different signal types (T – HbT; O – HbO; R – HbR; OR – relationship of oxy- and deoxyhemoglobin) are indicated by a left superscript to the variable of multifractal analysis.

^3^Note that the Bienaymé formula is not specific to fractal time series.

^4^Parameters obtianed directly from multifractal regression analysis are denoted as estimates with a hat: *H*(*q*) and ln(Ŝ_σ_(*N*)).

^5^low – right half (high frequencies) of the spectrum is excluded, w – windowing, e – endmatching.

^6^Since Wiener-Khinchin theorem only applies to wide-sense stationary processes, the autocorrelation function of the increment process is considered according to the fGn6fBm framework.

## Abbreviations

BOLD: blood oxygen level dependent
CBSI: correlation based signal improvement
Δ*H*_15_: the difference between the *H*(−15) and *H*(15) values
^*f*^: measure describing the fast signal component dominating over the low-frequency region
FMF: focus-based multifractal formalism (an approach using focus-based regression scheme)
*fwhm*: full-width of the singularity spectrum, *D*(*h*), at half of its maximum
*h*_max_: maximal Hölder exponent at which singularity strength (*D*) is equal to 1
LRC: long-range correlation
*N*_s_: number of non-overlapping segments
PSD: power spectral density
rsNIRS: resting-state near-infrared spectroscopy
*r*_σ_(*s*): scale-wise fractal correlation coefficient
^*s*^: measure describing the slow signal component dominant over the lower scales
*s*': scaling boundary (possible breakpoint)
^*X*^*S*_σ_(*q, s*): scaling function value at a given moment order (*q*) and temporal scale (*s*), calculated from signal *X*, with σ as measure
ln(^*X*^Ŝ(*N*)): the focus of the scaling function for signal *X*
SR: scaling range
SSC: signal summation conversion (method)
SSE: sum of squared error
*v*: the order of non-overlapping segments *v* = 1, …, *N*_s_
(V)LFO: (very) low-frequency oscillation.
