# Supplementary material for: Impact of Healthy Aging on Multifractal Hemodynamic Fluctuations in the Human Prefrontal Cortex
Source: Front Physiol. 2018 Aug 10;9:1072. doi: 10.3389/fphys.2018.01072 (PMC6097581; doi:10.3389/fphys.2018.01072)
Supplement: Supplementary file 1 [file Presentation_1.pdf]

Supplementary Material

Impact of Healthy Aging on Multifractal Hemodynamic Fluctuations in the Human Prefrontal Cortex

Peter Mukli, Zoltan Nagy[#], Frigyes Samuel Racz[#], Peter Herman and Andras Eke*

*** Correspondence:** Andras Eke: eke.andras@med.semmelweis-univ.hu

# Equally contributing authors.

1 *In silico* experiments to substantiate attenuated hemodynamics

As we specifically instructed our subjects to refrain from head movements, our raw signals must have contained a mixture of systemic cardiovascular, local vascular and local neural contributions. In terms of the relationship between oxy- (HbO) and deoxyhemoglobin (HbR), either correlated or anticorrelated fluctuations are elicited by these influences. The purpose of correlation-based signal improvement (CBSI) — the preprocessing step used in this study — is to enhance the separation of neurogenic and vasogenic fluctuations based on their differing influence on the strength of HbO-HbR relationship. At this end, our aim in these *in silico* experiments was to assess their cross-correlation during a "perturbation" (representing functional hyperemia) for enhancing the interpretation of the relative and time-varying impact of oxygen extraction (due to neural activity), local and systemic vascular effects.

The model of Buxton et al. (Buxton et al., 1998) treats the regional vascular compartment (i.e. the one being connected to a perforating artery in the brain cortex) as a "balloon" with given viscoelastic properties. The actual size of the balloon is determined by the balance between its arterial blood inflow, $f_{in}(t)$, and venous blood outflow, $f_{out}(t)$. The latter at constant cerebral oxygen consumption — via the oxygen content of blood within the balloon — will determine the time course of oxygen-dependent modalities such as BOLD (blood oxygen level dependent signal) (Buxton et al., 1998) or for that matter HbO and HbR signals. This model found widespread use in brain activation studies for describing the hemodynamics during periods of transient hyperperfusion (i.e. functional hyperaemia) as captured by fMRI-BOLD or fNIRS. Cui et al. (Cui et al., 2010) used this model for simulating the link between balloon dynamics and running correlation (Pearson, t=5 s) between HbO(t) and HbR(t) measured by NIRS during transient hyper- and hypoperfusion elicited by passive head movements.

In this study, we performed *in silico* hemodynamic experiments based on the balloon model of Buxton et al. (Buxton et al., 1998) in order to substantiate our *in vivo* findings with $r(s)$ difference observed between young and elderly group measurement groups. Hemodynamic response to neural

activity was treated as an input of the balloon model simulated by transients in blood inflow, (f_{in}). $f_{in}(t)$ can be described in a combination of relative baseline (f_0), direction and magnitude (Δf) of change as

$$f_{in}(t) = f_0 + \Delta f \left(\frac{t}{t_p} e^{1-\frac{t}{t_p}} \right)^{\delta}. \quad \text{(S1)}$$

The ascending and descending part of $f_{in}(t)$ was modelled with a gamma variate function (GVF), where t refers to real time, t_p denotes the peak time of the GVF and δ is a dispersional parameter set to 5.21 in the entire *in silico* study (Herman et al., 2009). The duration of the transient is determined by t_p, which was set to 1.8 s in all simulations. Values to Δf and t_p were assigned to achieve a reasonable match the CBF response to a step change in arterial blood pressure decaying in about 5 seconds (Aaslid et al., 1989). The effect of perturbed $f_{in}(t)$ on balloon-level hemodynamics was investigated for a single-cycle response within the time frame of simulation starting with zero time, t_0 (**Figure S1**). The balloon properties are described by two RC-components (representing resistance and compliance) with a characteristic time (τ) describing transit (subscript $_0$) and "post stimulus undershoot" (subscript $_v$). By solving the differential equation system (**Eqs. S2-S4**), the dynamics of oxygen extraction fraction (E) from the balloon, HbO and HbR were obtained as predictions of these *in silico* experiments.

$$\dot{q}(t) = \frac{f_{in}(t)}{\tau_0}\left[\frac{E(t)}{E_0} - \frac{q(t)}{v(t)}\right] + \frac{1}{\tau_v}\left[f_{in}(t) - v^{\frac{1}{\alpha}}\right]\frac{q(t)}{v(t)},$$

$$\dot{v}(t) = \frac{1}{\tau_v}\left[f_{in}(t) - v^{\frac{1}{\alpha}}\right], \quad \text{(S2-S4)}$$

$$\dot{p}(t) = \frac{1}{\tau_v}\left[f_{in}(t) - v^{\frac{1}{\alpha}}\right]\frac{p(t)}{v(t)}.$$

A testing framework was designed to evaluate the influence of baseline perfusion, frequency of hemodynamic response and viscoelastic properties on the HbO-HbR cross-correlation. The relative values chosen for f_0 were: 0, 0.3 and 1 corresponding to zero, low or normal baseline flow, respectively. The balloon dynamics was analyzed for periodic times (T) set to 12, 20, 60, 300 and 1200 seconds. The hemodynamic transient brought about by neurovascular coupling was altered by varying τ_0 and τ_v (see **Table S1**) representing RC-elements in the model. From the estimates of HbO and HbR, the *in silico* $r(T)$ — the endpoint of these simulations obtained at balloon cycle duration — was calculated in the same way as $r_\sigma(s)$ from the measured *in vivo* HbO and HbR records for given time scales. Please note that these simulations correspond to single-balloon dynamics for a single cycle, while the *in vivo* observed $r_\sigma(s)$ reflect spatial average of multiple cycling for multiple balloons in the ROI at various time scales.

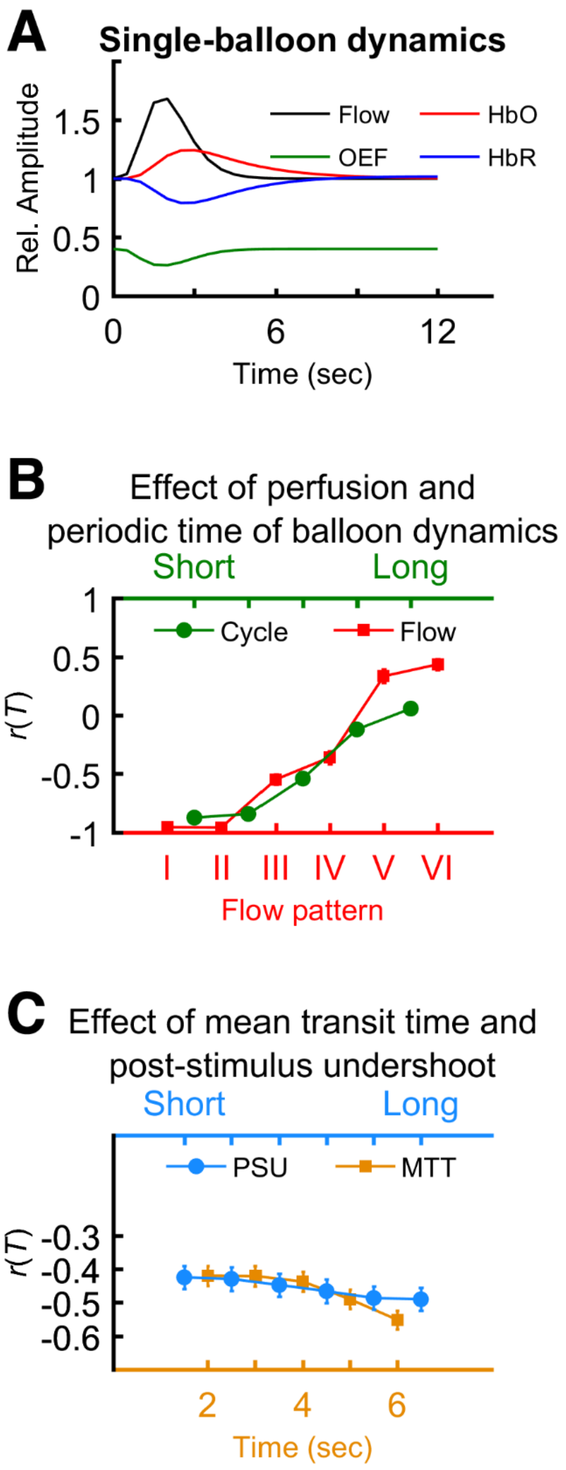

Figure S1. *Simulation of HbO-HbR relationship during functional hyperemia.* Single-balloon dynamics (**A**) is shown in terms of changes in inflow (Eq. S1). Oxygen extraction fraction, HbO and HbR were calculated from Eq. S2-S4. Flow pattern-dependent increase in perfusion (red) or length of cycling period (green) fundamentally affecting the cross-correlation of hemoglobin chromophores (**B**)

may well implicate attenuated vascular dynamics (for parameter values see **Table S1**). Please note that increased periodic time, T, represents a case of decreased incoming signalling. The effect of time constants of the model (post-stimulus undershoot, mean transit time) is moderate but also unequivocal (**C**) supporting a possible contribution of vascular stiffening or endothelial dysfunction to the observed hemodynamics.

In **Figure S1, panel B** we show predictions for $r(T)$ for different baseline flow amplitudes and balloon cycling frequencies. Even healthy aging may implicate a drop in regional perfusion; accordingly, on the one hand a reduced f_0 results in a marked increase in HbO-HbR correlation, despite the increased E, which is an anticorrelating effect. On the other hand, decreased cycling frequency is also accompanied with increased $r(T)$, which could be explained by the decreased triggering rate of neurovascular coupling likely due to decreased incoming (neural) signalling. Together, these results indicate that the HbO-HbR correlation dynamics is mainly determined by the degree of exchange in the balloon compartment: relatively deoxygenated blood is continuously replenished by oxygen-rich blood via $f_{in}(t)$ thus anticorrelating the HbO-HbR dynamics, which is further enhanced by transients due to hemodynamic response.

Table S1. Balloon model parameters in our testing framework

**Flow pattern**	**Varying parameters of Eq. S1**
I	$f_0 = 1$, Δf=+0.7
II	$f_0 = 1$, Δf=+0.2
III	$f_0 = 0.3$, Δf=+0.7
IV	$f_0 = 0.3$, Δf=+0.2
V	$f_0 = 0$, Δf=+0.7
VI	$f_0 = 0$, Δf=+0.2

**Cycling period**	**Short**					**Long**
12	18	21	24	27	30	33
20	20	25	30	35	40	45
60	20	30	40	50	60	70
300	30	60	90	120	150	180
l200	60	120	180	240	300	360

Modifying balloon viscoelastic properties — with maintained f_0 and cycling periods[1] — also moderately alters the observed $r(T)$ (**Figure S1, panel C**). Increased τ_0 and τ_v imply a lengthening transit of blood through the balloon allowing more time for oxygen extraction, which renders the HbO-HbR relationship more anticorrelated. Decreased mean transit time and post stimulus undershoot are associated with decreased compliances responsible for more rapid transient upon perturbation in $f_{in}(t)$ allowing less time for oxygen extraction. Hence it is reasonable to regard an increase in $r(T)$ as a manifestation of either vascular stiffening or dysfunctional endothelium.

Symbols and abbreviations for the Balloon model

q — deoxyhemoglobin

v — blood volume

p — total hemoglobin

f_{in} – blood inflow

E — oxygen extraction fraction

E_0 — resting net oxygen extraction fraction (Buxton et al., 2004)

τ_0 — mean transit time

τ_v — duration of post-stimulus undershoot (Mildner et al., 2001)

α — "fitted flow-volume coefficient" as described by Grubb et al. (Grubb et al., 1974)

2 *In silico* experiments to substantiate attenuated neurodynamics

Our rsNIRS recording via neurovascular coupling captures the hemodynamic fingerprint of a hierarchical neural system having multiple inputs at local neuronal levels (ion channels — integrate and — fire) and a single output converging as incoming signalling at the ROI level (Freeman et al., 2003), each exhibiting scale-free correlations (Werner, 2010). Consistent with this view, the incoming network size and associated signalling were simulated using the sand pile square lattice model of Bak et al. (Bak et al., 1987), where the size of the network was defined by the size of the lattice and the resting state incoming signaling by the random dropping of sand grains integrated as global response over the lattice as follows.

[1] Only main effects were evaluated in the statistical analysis, interaction between model parameters (treated as independent variables) were not taken into account.

We carried out *in silico* experiments in addition to demonstrate the link between global temporal correlation, $\hat{H}(2)$ and focus (maximal variance, $\ln(\hat{S}_{\sigma}(N))$) to substantiate the observed decrease in, $\hat{H}(2)$, and focus as manifestations of an age-related decline in incoming signaling. To simulate regional temporal neurodynamics, we relied on a modified version of the cellular automaton square lattice model (Bak et al., 1987) to allow its use in a small-world setting known to be a fundamental feature of the human cerebrocortical neural network (Sporns, 2006). To employ cellular automata with a small-world instead of the regular lattice connection layout, adjacency matrices of small-world networks were created according to the Watts-Strogatz method (Watts and Strogatz, 1998) that were used as the connection layout of the automata. The generator parameters were the number of nodes in the graph representing network size, *NS* [100, 400, 900, 1600], the node degree, defining the number of links and therefore the connection density, *D*, the fraction of existing edges to all possible edges in the network, [<0.1, 0.2, 0.3, 0.4, 0.5]. Both parameters reflect upon interrelated aspects of the input network, specifically, size and cross-scale interactions. The edge randomization parameter p was set between 0.015 and 0.03, yielding networks with high clustering coefficient while still having low average shortest path length, as per the definition of small-world topology by Watts and Strogatz (1998). Continuous external perturbation (one sand grain falling on a randomly chosen cell per cycle) established a self-organized critical state while the system is observed for $>2^{18}$ cycles in this state: the number of sand grains in the system is captured in $F(t)$. Each node has a critical value determined by its node degree: when such number of sand grain accumulates at that node relaxation occurs. At this point grains get equally distributed among the directly connected nodes giving rise to avalanches of various sizes in the system. 20 realizations were produced for every case of density and network size.

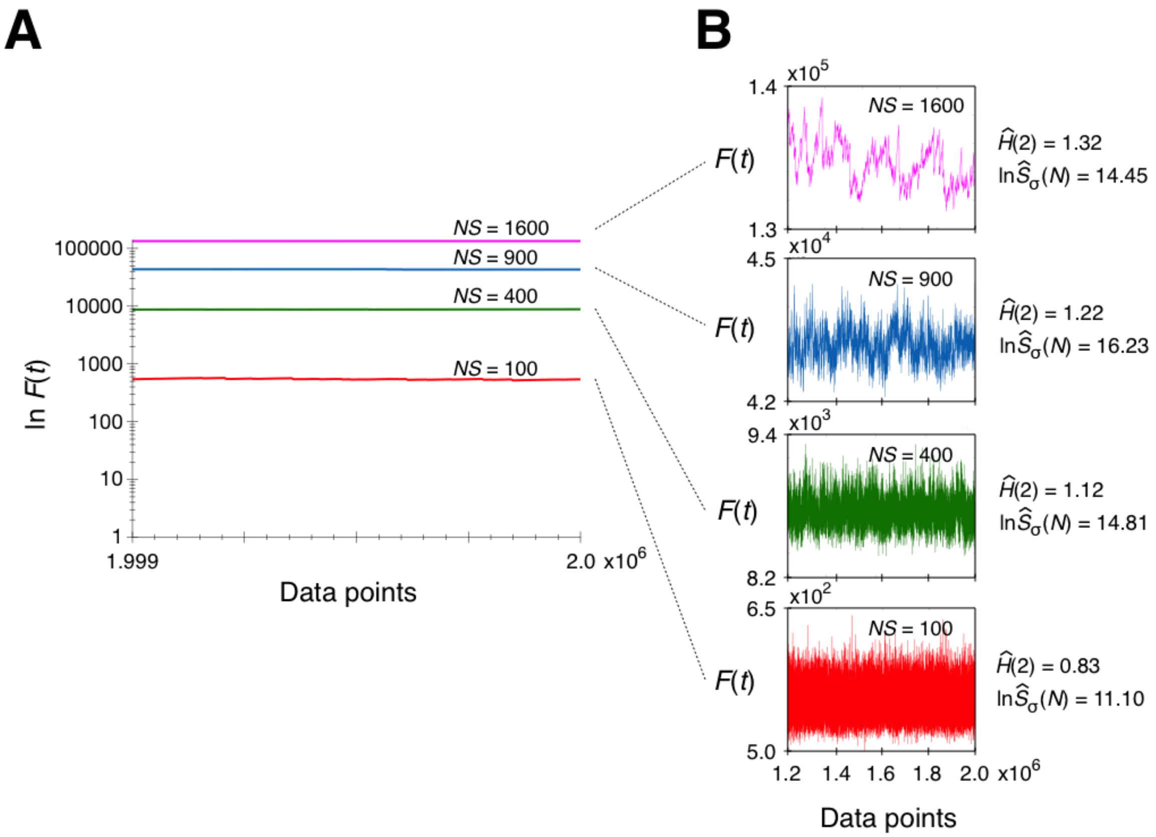

Figure S2. *Global scale-free temporal dynamics generated by sand pile model in cellular automata with small-world connectivity structures.* In the critical state, the number of sand grains in the system (**A**) is determined by the relationship between the number of grain leaving the system at the edges during network avalanches and the number of grain falling in the network due to external perturbation. The temporal structuring of these signals is shown in (**B**) — where amplitude is rescaled to match each signal's fluctuation range — along with displaying their associated $\hat{H}(2)$ and $\ln(\hat{S}(N))$.

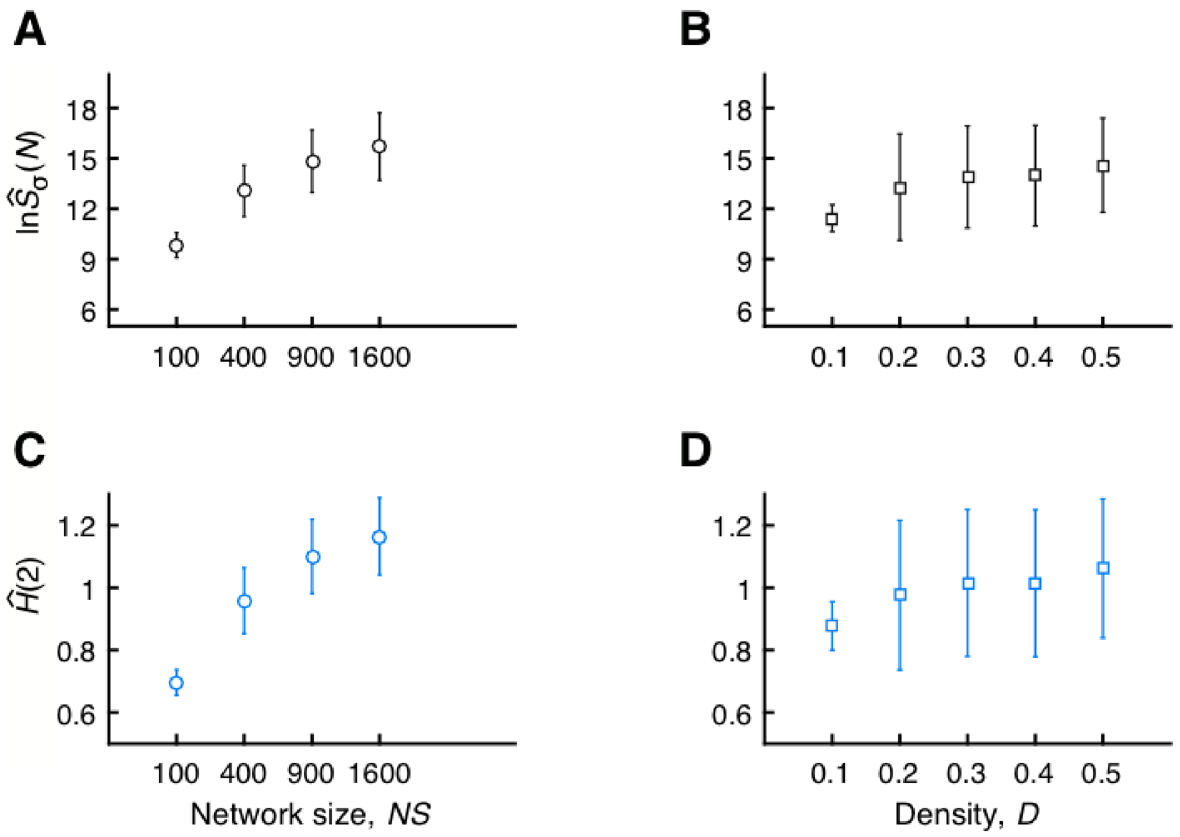

Figure S3. *Relationship between endpoint parameters of multifractal analysis and network metrics.* Focus of the global network dynamics (represented by $F(t)$) is shown as a function of network sizes (**A**) and densities (**B**). Multifractal analysis also yielded estimates of $H(2)$ with strikingly similar dependence on *NS* (**C**) and *D* (***D***). As larger network size and longer observation time allow for larger spatiotemporal scales over which component networks and their dynamics can assemble, the focus and the correlation of the input network dynamics captured in a single ROI will increase.

Multifractal analysis was performed at dyadic scales assuming a single scaling range (between s_{min}=4 and s_{max}=2048) and at q ranging from -15 to +15 with unit increments to evaluate the influence of network metrics on scaling properties at q=2 and estimated measure at s=N. The results of this analysis show that a decrease in network size is associated with a decreased magnitude of its global response to perturbation (related to $\ln(\hat{S}_\sigma(N))$ and decreased long-range correlation, $\hat{H}(2)$, too (**Figure S2, Figure S3, Panels A and C**). Next, we explored the relationship between connection density and the temporal correlations of global network dynamics, $\hat{H}(2)$ (**Figure S3, Panels B and D**) at the same range of *NS*. Notably, $\ln(\hat{S}_\sigma(N))$ and $\hat{H}(2)$ were found closely coupled across a wide range of link densities and network sizes that was reflected by the very similar dependence of these parameters as a function of either *NS* or *D*. Specifically, both of these parameters increased in case of larger number of nodes or higher network density. This suggests that these closely related aspects of network dynamics — ongoing intrinsic perturbation underlying resting-state activity — reflect upon network metrics.

Although based on measures of dynamics recorded from only a single ROI it is not possible to determine if network size or connection density changes, still inferences can be made on the incoming signalling as follows. The expected value of node degree corresponding to the ROI of our measurements is indeed the product of *NS* and *D*. Since both $\hat{H}(2)$ and $\ln(\hat{S}_\sigma(N))$ vary proportionally with *NS* and *D*, this nature of relationship should hold considering $NS \cdot D$ instead of *NS* or *D*.

This result of our *in silico* experiments (that is a model-based simulation of regional incoming neurodynamics) is consistent with the *in vivo* experimental findings of Baria et al. (Baria et al., 2013) evidencing that connectivity and complexity metrics are coupled. On this basis our interpretation of the age-related decrease in $\hat{H}(2)$ and $\ln(\hat{S}_\sigma(N))$ seems justified as a likely manifestation of age-related decline in neurodynamics resulting in decreased incoming signaling (concomitant to decreased node degree) (Meunier et al., 2009).

3 References

Aaslid, R., Lindegaard, K.F., Sorteberg, W., and Nornes, H. (1989). Cerebral autoregulation dynamics in humans. *Stroke* 20(1)**,** 45-52.

Bak, P., Tang, C., and Wiesenfeld, K. (1987). Self-organized criticality: An explanation of the 1/f noise. *Phys Rev Lett* 59(4)**,** 381-384. doi: 10.1103/PhysRevLett.59.381.

Baria, A.T., Mansour, A., Huang, L., Baliki, M.N., Cecchi, G.A., Mesulam, M.M., et al. (2013). Linking human brain local activity fluctuations to structural and functional network architectures. *Neuroimage* 73**,** 144-155. doi: 10.1016/j.neuroimage.2013.01.072.

Buxton, R.B., Uludag, K., Dubowitz, D.J., and Liu, T.T. (2004). Modeling the hemodynamic response to brain activation. *Neuroimage* 23 Suppl 1**,** S220-233. doi: 10.1016/j.neuroimage.2004.07.013.

Buxton, R.B., Wong, E.C., and Frank, L.R. (1998). Dynamics of blood flow and oxygenation changes during brain activation: The balloon model. *Magnetic Resonance in Medicine* 39(6)**,** 855-864. doi: 10.1002/mrm.1910390602.

Cui, X., Bray, S., and Reiss, A.L. (2010). Functional near infrared spectroscopy (NIRS) signal improvement based on negative correlation between oxygenated and deoxygenated hemoglobin dynamics. *Neuroimage* 49(4)**,** 3039-3046. doi: 10.1016/j.neuroimage.2009.11.050.

Grubb, R.L., Jr., Raichle, M.E., Eichling, J.O., and Ter-Pogossian, M.M. (1974). The effects of changes in PaCO2 on cerebral blood volume, blood flow, and vascular mean transit time. *Stroke* 5(5)**,** 630-639.

Herman, P., Sanganahalli, B.G., Blumenfeld, H., and Hyder, F. (2009). Cerebral oxygen demand for short-lived and steady-state events. *J Neurochem* 109 Suppl 1**,** 73-79. doi: 10.1111/j.1471-4159.2009.05844.x.

Meunier, D., Achard, S., Morcom, A., and Bullmore, E. (2009). Age-related changes in modular organization of human brain functional networks. *Neuroimage* 44(3)**,** 715-723. doi: 10.1016/j.neuroimage.2008.09.062.

Mildner, T., Norris, D.G., Schwarzbauer, C., and Wiggins, C.J. (2001). A qualitative test of the balloon model for BOLD-based MR signal changes at 3T. *Magn Reson Med* 46(5)**,** 891-899.

Sporns, O. (2006). Small-world connectivity, motif composition, and complexity of fractal neuronal connections. *Biosystems* 85(1)**,** 55-64. doi: 10.1016/j.biosystems.2006.02.008.

Watts, D.J., and Strogatz, S.H. (1998). Collective dynamics of 'small-world' networks. *Nature* 393(6684)**,** 440-442. doi: 10.1038/30918.

Werner, G. (2010). Fractals in the nervous system: conceptual implications for theoretical neuroscience. *Front Physiol* 1**,** 15. doi: 10.3389/fphys.2010.00015.
