# Supplementary material for: Impact of Healthy Aging on Multifractal Hemodynamic Fluctuations in the Human Prefrontal Cortex
Source: Front Physiol. 2018 Aug 10;9:1072. doi: 10.3389/fphys.2018.01072 (PMC6097581; doi:10.3389/fphys.2018.01072)
Supplement: Supplementary file 2 [file Data_Sheet_1.pdf]

APPENDIX

Evaluation of true multifractality and bimodality

General scale-free behavior

General fractal nature can be identified in the frequency domain, where it manifests as a scale-free structuring of the power spectrum (Eke et al., 2000; Eke et al., 2002) (**Figure A1B**). The presence of power-law scaling should be verified prior to fractal analysis (Eke et al., 2002) within the actual SR of each time series and was identified following the recommendations of Clauset et al. (Clauset et al., 2009). β of each time series was estimated with the ${}^{low}PSD_{w,e}$ method (Eke et al., 2000). Then, n=100 power-law distributed data sets with matching β and length were generated with the spectral synthesis method (Saupe, 1988). The p-value was defined as the fraction of the surrogate datasets that show better fit to a power-law function than the original time series (using the Kolmogorov-Smirnov statistics). A time series was considered scale-free in the case of p>0.1. Accordingly, we performed the statistical tests and analyses proposed by Clauset et al. on the corresponding spectral estimates (Clauset et al., 2009). This approach is generally applicable on apparently scale-free data of any kind (for example see these Refs. (Lo et al., 2002; He, 2011; Ihlen and Vereijken, 2013)). In our case it revealed that the power-law model failed on the data of eight subjects (**Table A1**) who thus were excluded from further analysis.

Segregating true multifractality from multifractal noise

Quantification of this *pseudomultifractality* (arising from multifractal noise, see (Grech and Pamuła, 2012) can be carried out by reshuffling techniques such as the procedures applied to rainfall data (Roux et al., 2009) or response time series (Ihlen and Vereijken, 2010). These methods preserve correlation structuring in the signal while eliminating local singular behaviors giving rise to multifractality. Alternatively, the supporting base of $D(h)$ can be used to determine if the analyzed signal is mono- or multifractal, as it was shown from electroencephalograms (Dutta, 2010; Gomez-Extremera et al., 2016). In this paper, an adaptive testing framework was elaborated similar to the latter approach following the methodological steps proposed in Refs. (Schumann and Kantelhardt, 2011; Grech and Pamuła, 2012). Multifractal estimates presented in the paper of Grech and Pamuła, in fact, have a much narrower supporting base as those of true multifractals, which served as one of the means for their segregation (Schumann and Kantelhardt, 2011; Grech and Pamuła, 2012). Given the deterministic relationship between $H(q)$ and $D(h)$, we applied the concept of supporting base – originally defined for $D(h)$ – to $\hat{H}(q)$. Hence, the presented adaptive "mono- *versus* multifractal contrast" test focusing on the range of $H(q)$ confirms if the supporting base of the empirical signals exceeds that of a multifractal noise (**Figure A1C**) (Racz et al., 2018). Accordingly, for every time series, n=100 monofractal (multifractal noise) signals with same length and $H(2)$ were generated with the Davies-Harte method (Davies and Harte, 1987) and analysed with the FMF-SSC method. Then, one sample t-test or Wilcoxon rank sum test was performed at α_s=0.05

(level of significance) to check if the ΔH_{15} values obtained from surrogate datasets were in fact smaller than that of the original time series. In fact, the more divergently the variance profiles fan out from their focus the higher is the possibility that the signal is a true multifractal reflected by an increased ΔH_{15}. This test verified that all subject's NIRS signals were true multifractals as the difference in the scaling of fluctuations of different sizes were significantly larger than those of *pseudomultifractal* noise signals with same monofractal property.

Distribution- versus correlation-type multifractality

In order to ensure that the origin of scale-invariance is due to genuine autocorrelation in the signal and not a broad power-law probability density function of its values (Kantelhardt et al., 2002; Eke et al., 2012), each time series was shuffled and then re-analysed in n=100 realizations, as shuffling destroys LRC but has no effect on the distribution (**Figure A1D**). Since LRC is attributed to q=2 (and fractal analysis is precise for small positive moments), $\hat{H}(2)$ of the shuffled time series were compared to that from the original time series with one sample t-test or Wilcoxon rank sum test at α_s=0.05. A time series was considered LRC multifractal, if shuffling significantly decreased estimated $H(2)$ towards 0.5. While shuffling procedure destroyed the LRC structuring enabling to segregate cases of distribution-type fractality, none of the signals proved to be falling in this category.

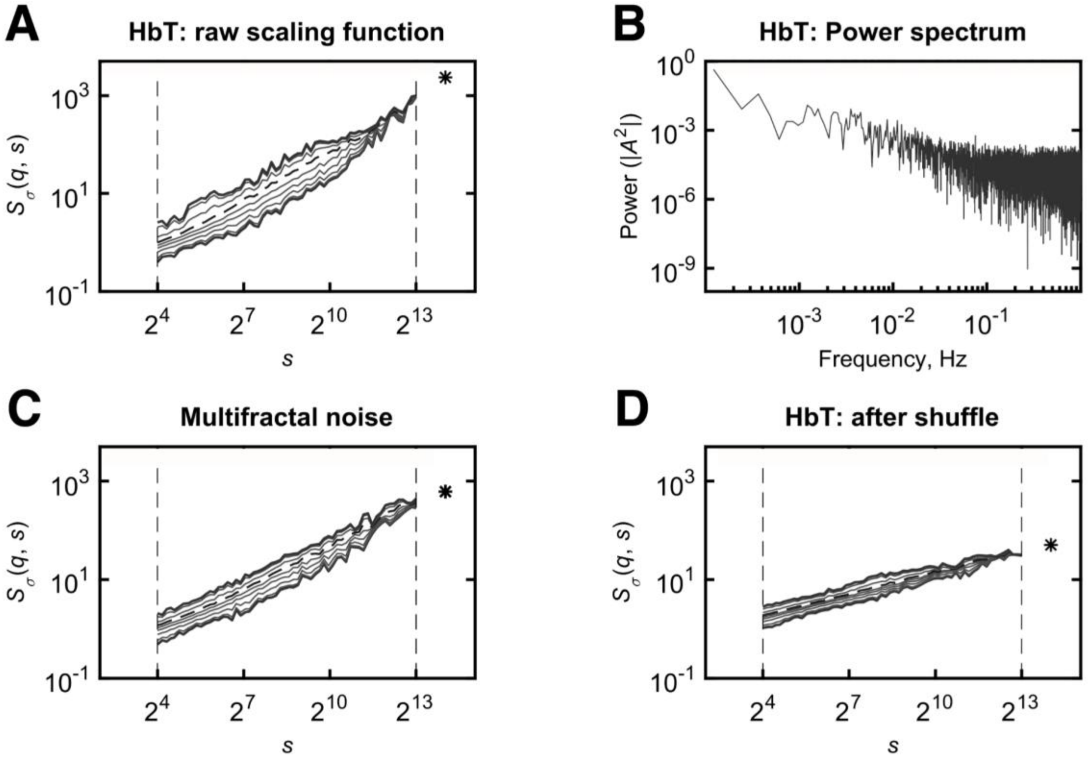

Figure A1. *Verification of scale-free property and characterization of multifractality in terms of noise-, distribution and correlation-type multifractality.* (**A**) Scaling function, $S_\sigma(q, s)$ of HbT signal; s: scale, q: moment order. (**B**) Power spectrum of HbT signal. A: amplitude. (**C**) Scaling function of monofractal signal generated at equal length (N) and $H(2)$. While Grech and Pamuła used an empirical level for separating mono- and multifractals (given as 0.12 for $N=2^{14}$), our method adjusts the process to $\hat{H}(2)$ of the signal in question. (**D**) Scaling function of shuffled HbT signal. Note that in case of empirical multifractal signals, both distribution- and correlation-type multifractality are usually present to some extent, therefore as shuffling will reduce $\hat{H}(2)$ of a mainly correlation-type multifractal, $\hat{H}(2)$ would not reach the theoretical 0.5 value of fully uncorrelated time series.

Table A1. Excluded subjects – Subject_{ID} (age)

Power-law test			Multifractal noise test			LRC test		
HbO	HbR	HbT	HbO	HbR	HbT	HbO	HbR	HbT
F_{21} (56) M_{34} (27)	F_{12} (46) F_{20} (56) M_{33} (25)	M_{40} (39) M_{42} (45)		none			none	

Screening for bimodal character of the measured resting state NIRS signals

Bimodality was evaluated in all of the cases remaining by performing *F*-test on the ratio of model-misfits normalized by the number of model parameters as given by

$$F = \frac{\left(\frac{SSE_1 - SSE_2}{p_2 - p_1}\right)}{\left(\frac{SSE_2}{n - p_2 - 1}\right)}, \tag{A1}$$

where subscript 1 refers to the unimodal fit (with no breakpoints and lower number of parameters) while subscript 2 refers to the scaling-range adaptive bimodal fit. Accordingly, p_1 is the number of model parameters (number of used moment orders and focus) for the unimodal regression analysis, while p_2 is the same for the bimodal regression analysis. Estimation was based on n=1860 scaling function values as the product of the number of moment orders (31) and scales used (60); meanwhile: p_1=32 and $p_2 = 2p_1 = 64$.

All scaling functions seemed having a bimodal structure with a two-teared scaling of apparent power-law characteristics separated either by a convex or concave transient range (27 % and 73 % of all cases, respectively). Eight of fifty-two subjects were excluded from the study based on their low ΔH_{15} (estimated for either of HbO, HbR and HbT) indicating multifractal noise. No further data were discarded based on shuffle-test or power-law test. In the majority of evaluated cases bimodality of scaling function was confirmed. However, two more subjects were excluded due to unacceptable fit of the bimodal model. Thus 42 subjects were promoted to group-level statistical analysis (**Table A1**). Of note, after the removal of these outliers, all $\hat{H}(2)$ and h_{max} samples were normally distributed permitting of parametric tests (such as two-way ANOVA).

References

Clauset, A., Shalizi, C.R., and Newman, M.E.J. (2009). Power-law distributions in empirical data. *SIAM Review* 51(4)**,** 661-703. doi: 10.1137/070710111.

Davies, R.B., and Harte, D.S. (1987). Tests for hurst effect. *Biometrika* 74(1)**,** 95-101. doi: 10.1093/biomet/74.1.95.

Dutta, S. (2010). Eeg Pattern of Normal and Epileptic Rats: Monofractal or Multifractal? *Fractals* 18(4)**,** 425-431. doi: 10.1142/S0218348x10005081.

Eke, A., Hermán, P., Bassingthwaighte, J., Raymond, G., Percival, D., Cannon, M., et al. (2000). Physiological time series: distinguishing fractal noises from motions. *Pflügers Archiv - European Journal of Physiology* 439(4)**,** 403-415. doi: 10.1007/s004249900135.

Eke, A., Herman, P., Kocsis, L., and Kozak, L.R. (2002). Fractal characterization of complexity in temporal physiological signals. *Physiol Meas* 23(1)**,** R1-38.

Eke, A., Herman, P., Sanganahalli, B.G., Hyder, F., Mukli, P., and Nagy, Z. (2012). Pitfalls in fractal time series analysis: Fmri BOLD as an exemplary case. *Front Physiol* 3 NOV. doi: 10.3389/fphys.2012.00417.

Gomez-Extremera, M., Carpena, P., Ivanov, P.C., and Bernaola-Galvan, P.A. (2016). Magnitude and sign of long-range correlated time series: Decomposition and surrogate signal generation. *Phys Rev E* 93(4). doi: ARTN 042201
10.1103/PhysRevE.93.042201.

Grech, D., and Pamuła, G. (2012). Multifractal background noise of monofractal signals. *Acta Phys Pol* 121(2 B)**,** B34-B39. doi: 10.12693/APhysPolA.121.B-34.

He, B.J. (2011). Scale-free properties of the functional magnetic resonance imaging signal during rest and task. *J Neurosci* 31(39)**,** 13786-13795. doi: 10.1523/JNEUROSCI.2111-11.2011.

Ihlen, E.A., and Vereijken, B. (2010). Interaction-dominant dynamics in human cognition: beyond 1/f(alpha) fluctuation. *J Exp Psychol Gen* 139(3)**,** 436-463. doi: 10.1037/a0019098.

Ihlen, E.A., and Vereijken, B. (2013). Multifractal formalisms of human behavior. *Hum Mov Sci* 32(4)**,** 633-651. doi: 10.1016/j.humov.2013.01.008.

Kantelhardt, J.W., Zschiegner, S.A., Koscielny-Bunde, E., Havlin, S., Bunde, A., and Stanley, H.E. (2002). Multifractal detrended fluctuation analysis of nonstationary time series. *Physica A* 316(1-4)**,** 87-114. doi: 10.1016/S0378-4371(02)01383-3.

Lo, C.C., Amaral, L.A.N., Havlin, S., Ivanov, P.C., Penzel, T., Peter, J.H., et al. (2002). Dynamics of sleep-wake transitions during sleep. *Europhys Lett* 57(5)**,** 625-631. doi: DOI 10.1209/epl/i2002-00508-7.

Racz, F.S., Mukli, P., Nagy, Z., and Eke, A. (2018). Multifractal dynamics of resting-state functional connectivity in the prefrontal cortex. *Physiol Meas*. doi: 10.1088/1361-6579/aaa916.

Roux, S.G., Venugopal, V., Fienberg, K., Arneodo, A., and Foufoula-Georgiou, E. (2009). Evidence for inherent nonlinearity in temporal rainfall. *Adv Water Resour* 32(1)**,** 41-48. doi: 10.1016/j.advwatres.2008.09.007.

Saupe, D. (1988). "Algorithms for random fractals," in *The Science of Fractal Images,* eds. H.-O. Peitgen & D. Saupe. (New York: Springer-Verlag), 71-136.

Schumann, A.Y., and Kantelhardt, J.W. (2011). Multifractal moving average analysis and test of multifractal model with tuned correlations. *Physica A* 390(14)**,** 2637-2654. doi: 10.1016/j.physa.2011.03.002.
